# A novel trivalent non-Fc anti-CD3 Collabody preferentially induces Th1 cell apoptosis *in vitro* and long-lasting remission in recent-onset diabetic NOD mice

**DOI:** 10.3389/fimmu.2023.1201853

**Published:** 2023-08-03

**Authors:** Chuan-Chuan Huang, Hsiang-Hsuan Sung, Hsiu-Chuan Li, Shi-Chuen Miaw, John T. Kung, Min-Yuan Chou, Betty A. Wu-Hsieh

**Affiliations:** ^1^ Graduate Institute of Immunology, National Taiwan University College of Medicine, Taipei, Taiwan; ^2^ Biomedical Technology and Device Research Laboratories, Industrial Technology Research Institute, Hsinchu, Taiwan; ^3^ National Laboratory Animal Center, National Applied Research Laboratories, Taipei, Taiwan; ^4^ Institute of Molecular Biology, Academia Sinica, Taipei, Taiwan

**Keywords:** CD3, Collabody, trivalent antibody, type 1 diabetes, Th1, Treg, apoptosis

## Abstract

Specific anti-CD3 treatment is deemed to be a promising therapy for allograft rejection and type 1 diabetes (T1D). Fc receptor (FcR) reduced-binding antibodies, by avoiding adverse effects of Fc and FcR interaction, have good therapeutic potential. We generated a trivalent anti-mouse-CD3 Collabody, h145CSA, by using a triplex-forming collagen-like peptide (Gly-Pro-Pro)_10_ to drive the trimerization of the Fab fragments. Exposure to h145CSA, but not its bivalent counterparts 145-2C11 and h145chIgGAA (FcR reduced-binding format), upregulates FasL expression on Th1 cells and causes Th1 cell apoptosis. Administration of h145CSA invokes minimal mitogenic effects in mice. The ability of multiple dosing of h145CSA to induce splenic CD4^+^ T-cell depletion is comparable to bivalent antibodies but is characterized by more rapid CD4^+^ T-cell recovery kinetics. h145CSA is more potent than h145chIgGAA in inducing long-lasting remission in recent-onset diabetic NOD mice. Its therapeutic effect is accompanied by a significantly lower percentage of CD4^+^IFNγ^+^ T cells and a higher Treg/Th1 ratio in pancreatic and mesenteric lymph nodes. The results of our study demonstrate that trivalent non-Fc anti-CD3 Collabody has the potential to be used in the treatment of T1D.

## Introduction

T-cell-mediated immunity is crucial in the development of autoimmunity. Antibody therapy directly targeting the CD3 molecule of the TCR complex abrogates pathogenic T cells and/or modulates T-cell response and is deemed a promising approach to treating autoimmune disease. Muromonab (OKT3), a murine antibody recognizing the human CD3*ε* chain, was the first monoclonal antibody (mAb) approved by US FDA to be used clinically (1). A modified version of OKT3, Teplizumab, was recently approved for the treatment of individuals at high risk for type 1 diabetes (T1D).

Anti-CD3 antibodies are known for their immunomodulatory effects. CD3-specific antibodies binding to CD3/TCR complex induce internalization or shedding of the complex, resulting in T cells being “blind” to antigens (2). Induction of apoptosis is another mechanism by which anti-CD3 antibodies eliminate activated T cells (3, 4). Different T-cell subsets differ in their responsiveness to anti-CD3 antibody treatment. CD4^+^FoxP3^+^ regulatory T cells, contrasting to Th1 cells, are more resistant to anit-CD3 antibody-induced cell death (5). By selectively depleting pathogenic Th1 cells but preserving Treg cells, anti-CD3 antibody treatment promotes immune tolerance (5). Clinical studies of anti-CD3 therapy in patients with recent-onset T1D showed that the therapy induces a population of regulatory/exhausted CD8 T cells in responders (6, 7), suggesting that anti-CD3 antibody therapy may also function to regulate T-cell exhaustion in addition to Treg.

Clinical application of muromonab in solid organ transplantation is hampered by the first-injection syndrome resulting from T-cell activation and concomitant systemic release of cytokines, related complications, and, in severe cases, death (8, 9). T-cell activation induced by anti-CD3 monoclonal antibody depends on the interaction of the Fc portion of the mAb with Fc receptors (FcRs) on accessory cells, allowing multivalent crosslinking of the CD3/TCR complex (10). CD3-specific F(ab’)_2_ fragment, which lacks the Fc region, extended skin graft survival and prevented graft-versus-host disease (GVHD) lethality without evoking strong side effects in mouse models (11, 12). Another approach to avoiding Fc-mediated adverse effects is to introduce mutations into the CH_2_ domain at positions L234A/L235A (LALA) and N297A (13–16). These CD3-specific FcR reduced-binding mAbs have shown great promise in preclinical and clinical studies for the prevention of allograft rejection (13–16).

Humanized OKT3 antibodies with LALA mutations have been shown to reduce significantly but fail to eliminate binding to FcγRI and FcγRII (17, 18). The therapeutic efficacy of these FcR reduced-binding anti-CD3 mAbs was tested in autoimmune diseases. FcR reduced-binding anti-CD3 mAbs teplizumab and otelixizumab were used in phase I/II clinical trials to treat patients with recent onset of clinical T1D (19–23). Patients experienced T-cell activation, minor cytokine secretion, and Epstein–Barr virus reactivation (19, 22, 24). To avoid these adverse effects, antibody doses were reduced in the following phase III trial. However, treatment with a lower dose of anti-CD3 antibody did not meet the primary efficacy endpoint in patients with recent onset of clinical T1D (25, 26). In November 2022, US FDA approved the same low-dose treatment of teplizumab for individuals who are nondiabetic relatives of patients with T1D, have evidence of dysglycemia, and have two or more diabetes-related autoantibodies. Clinical trial results showed that teplizumab treatment successfully delayed the medium time to clinical T1D from 24.4 months (placebo group) to 48.4 months. However, teplizumab recipients experienced lymphopenia during the first 30 days but recovered after day 45. They also suffered Epstein–Barr virus reactivation from weeks 3 through 6. All recipients eventually recovered from the side-effects (27). These trials demonstrate that the therapeutic window of dose for effective anti-CD3 antibody treatment is narrow. The pharmacologically responsive high dose was effective but accompanied by adverse effects. Low-dose treatment, although it minimizes adverse effects, hinges on the choice of patient groups (recent onset vs. high risk yet clinically nondiabetic) (27, 28). Wilkinson et al. generated modified versions of OKT3 with specific amino acid substitutions in the Fc region at L234 and L235 combined with the substitution G236R (29). These variants show no detectable binding to Fc receptors. While the LALA variant induces one-third to one-half the amount of cytokines produced by wild-type OKT3, the modified versions of OKT3 with three substitutions do not (29). Therefore, we hypothesized that an anti-CD3 antibody that displays absolutely no FcR binding rather than weak binding that affords better safety profiles may be a viable option to widen the therapeutic window of anti-CD3 antibody therapies.

Collabodies are a class of multivalent protein binders that use a triplex-forming collagen-like peptide (Gly-Pro-Pro)_10_ to drive the trimerization of a target-binding domain. The antibody-binding domain is fused with a collagen-like domain to generate a collagen scaffold antibody (CSA). It can be successfully expressed as a soluble secretory protein in mammalian cells (30). The avidity effect of trivalent Collabody improves the dissociation rate (slower *k*_off_ rate) over its monovalent and bivalent counterparts upon binding to target molecules (30). The functional affinity of a trivalent anti-EGFR CSA (erb_scFv-Col) against human EGFR is 1,000- and 20-fold higher than its monovalent and bivalent counterparts, respectively. Compared to bivalent IgG, human CD3*ε*-specific CSA is more effective in inhibiting anti-CD3 IgG binding to T cells and induces a greater degree of CD3/TCR downregulation from the cell surface, a mechanism that suppresses T-cell immunity (30). Compared to bivalent antibodies, trivalent anti-CD3 Collabody may transduce a stronger TCR signal due to its higher valency. Additionally, Collabody non-Fc structure can avoid both higher-order crosslinking of CD3/TCR complex and induction of T-cell activation by Fc binding to cells expressing FcR. These characteristics makes Collabodies potentially advantageous for therapeutic purposes.

In this study, we generated and characterized an anti-mouse CD3 Collabody, h145CSA. The initial characterizations of h145CSA were performed in healthy C57BL/6 and BALB/c mice for the consideration of its potential wide application. The *in vitro* and *in vivo* biological functions of h145CSA were compared to their bivalent counterparts. h145CSA was more potent in inducing Th1 cell apoptosis than its bivalent controls. Administration of multiple doses of h145CSA induced T-cell depletion in the spleen as efficiently as did other bivalent anti-CD3 antibodies. However, T-cell recovery kinetics was faster in mice receiving h145CSA treatment than in those receiving bivalent anti-CD3 antibodies. In addition, h145CSA was active *in vivo* with minimal mitogenic side-effects. NOD mice were used to explore whether h145CSA is efficacious in reverting T1D. Results showed that h145CSA treatment was more potent than FcR reduced-binding IgG (h145chIgGAA) in reverting diabetes in recent-onset diabetic NOD mice back to normoglycemia. Compared to h145chIgGAA treatment, NOD mice treated with h145CSA had a significantly lower percentage of CD4^+^IFNγ^+^ T cells and a higher Treg/Th1 ratio in pancreatic and mesenteric lymph nodes. Taken together, our results suggest that anti-CD3 Collabody possesses therapeutic potential for autoimmune diseases.

## Materials and methods

### Construction of recombinant plasmids

The *V_H_* and *V_L_* domains of the hamster anti-mouse CD3*ε* antibody (clone 145-2C11) were grafted onto their respective human CH_1_ and kappa light chain. To generate h145hIgG_1_, nucleotide sequences of the *V_H_* and *V_L_* regions of 145-2C11 were synthesized and cloned. *V_H_* was subcloned into mammalian expression vector pFUSE-CHIg-hIG_1_ (InvivoGen, San Diego, CA, USA), which harbors a signal peptide sequence, and *V_L_* into pSecTag2/Hygro (Invitrogen, Carlsbad, CA, USA), which contains the human kappa chain constant region. To make h145CSA, the coding regions of CH_2_ and CH_3_ were substituted by the peptide sequence EPKSCDKTHTCPPCPRSIP(GPP)_10_ *GICDPSLC*TG, which is composed of a hinge region of human IgG_1_ (underlined), a collagen-like peptide (GPP)10, and the NC1 disulfide knot region of type XXI collagen (in italics). To make chimeric IgG counterparts of h145CSA, the coding regions of the CH_2_ and CH_3_ domains of h145hIgG_1_ were replaced by those of murine IgG_2a_ with or without two alanine substitutions at amino acids L234 and L235 in the CH_2_ region using site-directed mutagenesis PCR, and they were designated h145chIgGAA and h145chIgG, respectively.

### Antibody expression and purification

All recombinant antibodies were obtained by stable transfection of expression constructs in Chinese hamster ovary (CHO-S) cells (Invitrogen) using Amaxa Nucleofector**^®^** kit V (Lonza Bioscience, Basel, Switzerland). Transfected cells were selected by culturing in 400 μg/ml hygromycin B (AG Scientific, San Diego, CA, USA) for 4 weeks. A stable clone was cultured in a chemically defined CD OptiCHO™ medium in a shaker flask at an initial seeding density of 3 × 10^5^ cells/ml. Cultures were terminated when cell viability dropped below 50%. Culture supernatants were collected and filtered. Fc-containing antibodies and h145CSA were purified by affinity resin MabSelect SuRe™ (Cytiva, Marlborough, MA, USA) and KappaSelect (Cytiva), respectively, which were equilibrated with 50 mM Tris-HCl buffer containing 0.15 M of NaCl (pH 8.0). The column was washed with equilibration buffer, followed by 2 M urea buffer containing 1.5 M NaCl, 5 mM EDTA, 50 mM l-histamine, and 50 mM Tris-HCl buffer containing 0.15 M of NaCl and 5% sucrose. The recombinant antibodies were eluted with 20.4 mM of phosphoric acid buffer containing 5% of sucrose (pH 2.5). The eluent was neutralized and dialyzed against DPBS (pH 7.0, Gibco, Waltham, MA, USA). The KappaSelect eluent of h145CSA was further purified by a Superdex 200 10/300 GL column (Cytiva) equilibrated with Dulbecco's phosphate-buffered saline (DPBS), and the trimer fraction was collected. To prepare h145F(ab**’**)_2_, h145hIgG_1_ was digested by pepsin in 50 mM sodium acetate buffer (pH 4.0) at a final enzyme:protein ratio of 1:20 (w/w) at 37°C for 8 h followed by size-exclusion chromatography using Superdex 200 10/300 GL column equilibrated with DPBS. Antibody concentration was determined by absorbance at 280 nm.

### Antibody characterization

Antibodies were analyzed in a 4%–12% NuPAGE Bis-Tris polyacrylamide gel (3 μg) and stained with InstantBlue Protein Stain. The purity and integrity of molecules (20 μg in 20 μl) were analyzed via size exclusion chromatography (SEC) using a Waters e2695 HPLC in combination with a Zenix-C SEC-300 column (3 μm, 7.8 mm × 3 mm, Sepax) at a flow rate of 1 ml/min using 1× PBS as mobile phase.

### Mice

**Figure 1:**
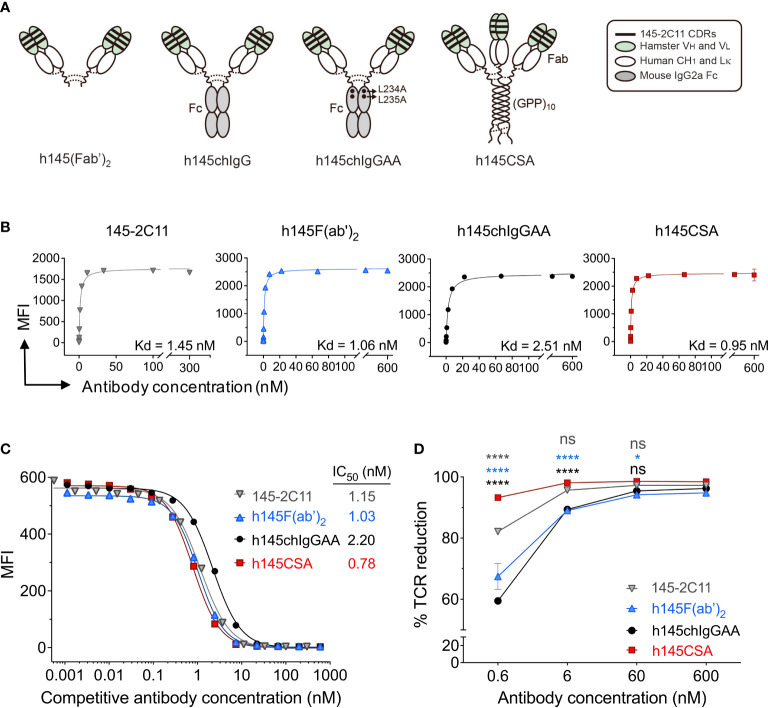
Characterization of h145CSA and its bivalent antibody counterparts. **(A)** Schematic illustration of different antibody formats. h145F(ab’)_2_ consists of the CH_1_ domain of human IgG_1_ and the human kappa light chain constant region fused with the *V_H_* and *V_L_* fragments of 145-2C11 antibody, respectively. The bivalent human/mouse chimeric IgG antibodies were generated by a fusion of the same Fab fragment as h145F(ab’)_2_ to the Fc domain of mouse IgG_2a_ with or without two alanine substitutions in the CH_2_ domain and named as h145chIgGAA and h145chIgG, respectively. h145CSA is composed of the same Fab fragment as h145F(ab’)_2_, followed by a collagen-like peptide (GPP)_10_ that is capable of self-trimerization into a triplex structure. Dotted lines indicate putative disulfide bonds. **(B–D)** Spleens from healthy female C57BL/6 mice at 6–12 weeks of age were collected. Mouse T cells were enriched by negative selection using mouse T-cell isolation kit. **(B)** Purified T cells were incubated with threefold serial dilutions of each anti-CD3 antibody ranging from 0 to 300 nM (145-2C11) or 600 nM (h145F(ab’)_2_, h145chIgGAA, and h145CSA) for 1 h on ice, followed by staining with FITC-conjugated goat anti-hamster IgG (145-2C11) or Alexa Fluor 647-conjugated goat anti-human IgG (H+L) (h145F(ab’)_2_, h145chIgGAA, and h145CSA). Mean fluorescent intensity (MFI) was analyzed by flow cytometry (*n* = 3). Kd was calculated by GraphPad Prism program 9.2.0. **(C)** Purified T cells were incubated with threefold serial dilutions of each anti-CD3 antibody on ice, followed by incubation with a subsaturating amount of Alexa Fluor 647-conjugated 145-2C11. After washing, bound Alexa Fluor 647-conjugated 145-2C11 on T cells was quantified by flow cytometry (mean MFI ± SEM, *n* = 3). IC_50_ values obtained from the inhibition curves were calculated by fitting nonlinear regression using GraphPad Prism program 9.2.0. **(D)** Purified splenic T cells were incubated with 10-fold serial dilutions of each anti-CD3 antibody at 37°C for 24 h Cells were stained with PE-conjugated anti-TCRβ antibody and subjected to flow cytometric analysis. The percentage of TCR downregulation = the MFI difference of TCR on the surface between antibody-treated and antibody-untreated T cells divided by the MFI of TCR on the surface of untreated T cells × 100. The results are expressed as the mean of triplicate experiments ± SEM.

Female C57BL/6 mice were purchased from the National Laboratory Animal Center (NLAC), NARLabs, Taiwan. All mice were maintained in the Laboratory Animal Center of Industrial Technology Research Institute (ITRI LAC) under specific pathogen-free (SPF) conditions. Healthy C57BL/6 mice at 6–12 weeks of age were used in experiments described in **Figures 1** **–** **6**.

For the pharmacokinetics study, BALB/c mice were obtained from BioLASCO (Taiwan) and kept in the Development Center for Biotechnology, Taiwan. Healthy male mice at 6–8 weeks of age were used. For the biodistribution study, BALB/c mice were purchased from NLAC Labs. Mice were kept in the Institute of Nuclear Energy Research, Taiwan. Healthy male mice at 6–8 weeks of age were used.

Female NOD/ShiLtJ and NOD/ShiLtJNarl mice were obtained from The Jackson Laboratory (Bar Harbor, ME) and NLAC, respectively. Therapeutic experiments assessing the therapeutic efficacy of h145chIgGAA and h145CSA on NOD/ShiLtJ mice were conducted by The Jackson Laboratory-Sacramento Facility *In Vivo* Service, California. Therapeutic experiments employing NOD/ShiLtJNarl mice were performed in NLAC, Taiwan.

Mice were euthanized by inhalation of carbon dioxide in the euthanasia chamber.

### Antibodies and reagents

Anti-CD3 (clone 145-2C11), anti-CD28 (clone 37.51), anti-IL-4 (clone 11B11), and anti-IFN-γ (clone XMG1.2) antibodies for T-cell activation and differentiation; anti-FasL (clone MFL3 and MFL4) blocking antibodies; staining antibodies Alexa Fluor 488-conjugated anti-CD4 (clone GK1.5), PE-conjugated anti-CD8 (clone 53-6.7), -TCRβ (clone H57–597), -Foxp3 (clone 150D), -CD69 (clone H1.2F3), PerCP/Cy5.5-conjugated anti-CD45 (clone 30-F11), Alexa Fluor 647-conjugated anti-IFN-γ (clone XMG1.2), -CD3 (clone 145-2C11), APC-conjugated -CD25 (clone 3C7), FITC-conjugated Annexin V, 7-AAD, biotin-conjugated anti-FasL (clone MFL3), and APC-conjugated streptavidin were all purchased from Biolegend, San Diego, CA, USA. Alexa Fluor 647-conjugated goat anti-human IgG (H+L) for T-cell binding assay was obtained from Thermo Fisher Scientific, Waltham, MA, USA. Fc Block was from Miltenyi Biotec, Bergisch Gladbach, North Rhine-Westphalia, Germany, and FACS staining buffer (BSA) was from BD Pharmigen, San Diego, CA, USA. Anti-human Fd-capturing antibody (clone HP6045) and horseradish peroxidase (HRP)-conjugated goat anti-human kappa light chain detecting antibody for ELISA were from Sigma-Aldrich, St. Louis, MO, USA and Bethyl Laboratories, Montgomery, TX, USA, respectively.

### T-cell enrichment

Spleen and lymph node cells were collected from healthy female C57BL/6 mice between 6 and 12 weeks of age. Total T cells and naïve CD4^+^ T cells were enriched using EasySep™ Mouse T-Cell Isolation Kit and Mouse Naïve CD4 T-Cell Isolation Kit (STEMCELL Technologies, Vancouver, Canada), respectively. Purified total T cells were used in direct and competitive T-cell binding and TCR downregulation assays. Naïve CD4^+^ T cells were used for activation and differentiation experiments.

### Staining and flow cytometry

To stain for cell surface antigens, total spleen cells were pretreated with Fc blocker before staining. Cells were then stained with fluorochrome-conjugated antibodies (anti-TCRβ, anti-CD4, anti-CD8, anti-CD69, and anti-CD25) and left on ice for 30 min before analysis. A high-sensitivity strategy was used to stain cell surface FasL as per the suggestion on the technical data sheet (BioLegend). To maximize the signal, cells were stained with biotin-conjugated anti-mouse FasL antibody (MFL3) and left at 4°C for 18 h, followed by staining with APC-conjugated streptavidin and left on ice for 1 h.

To stain Foxp3, cells were fixed and permeabilized with a True-Nuclear transcription factor buffer set (BioLegend) and stained with a PE-conjugated anti-Foxp3 antibody.

To detect intracellular IFN-γ, cells were stimulated with phorbol myristate acetate (PMA, 50 ng/ml)/ionomycin (500 ng/ml) in the presence of brefeldin A (10 μg/ml) for 4 h. Cells were fixed and permeabilized with True-Nuclear transcription factor buffer set (BioLegend) and stained with Alexa Fluor 647-conjugated anti-IFN-γ antibody. BD FACSCalibur™ (BD Biosciences) was employed for cell acquisition and FlowJo software for data analysis.

### Direct and competitive T-cell binding assays

Spleen and lymph node cells were collected from healthy female C57BL/6 mice between 6 and 12 weeks of age. Purified total T cells (1 × 10^5^ cells/tube) as described above were incubated with threefold serial dilutions of 145-2C11, h145F(ab’)_2_, h145chIgGAA, or h145CSA on ice for 1 h. To determine binding avidity, cells were washed with staining buffer and stained with FITC-conjugated goat anti-hamster IgG (for 145-2C11) or Alexa Fluor 647-conjugated goat anti-human IgG (H+L) (for h145F(ab’)_2_, h145chIgGAA, and h145CSA) and left on ice for 1 h prior to analysis by flow cytometry. The equilibrium dissociation constant (Kd) for each anti-CD3 antibody was calculated by fitting data points to a ligand binding algorithm *Y* = Bmax × *X*/(Kd + *X*), where *Y* is specific binding, Bmax is maximum specific binding, and *X* is ligand concentration, using GraphPad Prism version 9. To evaluate the effect of antibody valency on its dissociation rate, a competition binding assay was performed. T cells were incubated with threefold serial dilutions of 145-2C11, h145F(ab’)_2_, h145chIgGAA, or h145CSA on ice for 1 h. A subsaturating concentration (0.25 µg/ml) of Alexa Fluor 647-conjugated 145-2C11 antibody was added directly to the tube. After another 1 h of incubation, cells were washed with staining buffer and analyzed by flow cytometry. The inhibition curves were plotted by fitting data points to a nonlinear regression algorithm. The concentration of each mAb required to reduce 50% of the maximal fluorescence intensity (half maximal inhibitory concentration (IC_50_)) was obtained from the inhibition curve.

### TCR downregulation assay

Purified total T cells were plated at 5 × 10^4^ cells/well in a 96-well plate and incubated at 37°C in RPMI-1640 medium supplemented with l-glutamine, 1 mM sodium pyruvate, 50 µM β-mercaptoethanol, and 10% heat-inactivated fetal bovine serum (complete RPMI) in the presence of increasing concentrations of the 145-2C11, h145F(ab’)_2_, h145chIgGAA, or h145CSA for 24 h. Cells were washed, re-suspended in staining buffer, and stained with PE-conjugated anti-TCRβ mAb before being subjected to flow cytometric analysis.

TCR downregulation was calculated as it was described elsewhere (13):


$$\text{\% TCR downregulation}=100\times \frac{{\text{MFI}}_{\text{control cells}}-{\text{MFI}}_{\text{antibody}-\text{treated cells}}}{{\text{MFI}}_{\text{control cells}}}$$

Where MFI is the mean fluorescence intensity of stained cells.

### CD4^+^ T-cell activation and differentiation

To obtain nonpolarized activated CD4^+^, Th1, or Treg cells, naïve CD4^+^ T cells at 1 × 10^6^ cells/well were cultured in 48-well plates precoated with anti-CD3 antibody (2 μg/ml) for 2 days in complete RPMI medium containing soluble anti-CD28 antibody (2 μg/ml) in either nonpolarizing condition (100 U/ml IL-2), Th1-polarizing condition (100 U/ml IL-2, 10 ng/ml rIL-12 and 10 μg/ml anti-IL-4 antibody from clone 11B11), or Treg-polarizing condition (100 U/ml IL-2, 5 ng/ml TGF-β, 10 μg/ml anti-IL-4 antibody, and 10 μg/ml anti-IFN-γ antibody from clone XMG1.2). Nonpolarized activated CD4^+^ T cells, polarized Th1, or polarized Treg cells were harvested and re-plated onto non-anti-CD3 antibody-coated plates for another 2 days in either nonpolarizing, Th1-polarizing, or Treg-polarizing condition. Finally, nonpolarized activated CD4^+^, Th1, or Treg cells were harvested for further assays.

### *In vitro* T-cell apoptosis assay

Naïve CD4^+^ T, activated CD4^+^ T, Th1, and Treg cells (2 × 10^4^ cells/well) were incubated in a 96-well plate in the presence of serial logarithmic dilutions of 145-2C11, h145chIgGAA, or h145CSA for 6, 12, or 24 h. To inhibit Fas/FasL-mediated apoptosis, Th1 cells were cultured with 10 μg/ml of 145-2C11, h145chIgGAA, or h145CSA and serial 10-fold dilutions of the FasL-blocking mAb (clone MFL3 or MFL4). Cells were washed twice with cold DPBS and re-suspended in 100 μl of Annexin V binding buffer. After the addition of FITC-conjugated Annexin V and 7-AAD (5 µl each), cells were left at room temperature for 15 min in the dark, and analyzed by flow cytometry. Cell death was assessed by staining with FITC-conjugated Annexin V and 7-AAD. Annexin V^+^ cells (7-AAD^+^ or 7-AAD^−^) were defined as apoptotic cells. Percent-specific apoptotic cells were calculated as Δ Annexin V % = % Annexin V^+^ cells with anti-CD3 antibody treatment – % Annexin V^+^ cells in medium alone.

### Quantitative PCR

Total RNA was extracted with Quick-RNA™ MiniPrep kit (Zymo Research, Irvine, CA, USA). cDNA was reversely transcribed in a total volume of 20 μl reaction mixture containing RNA, first-strand buffer, DTT, dNTP, gene-specific reverse primers, SuperScript IV Reverse Transcriptase (Invitrogen), and RNaseOUT™ recombinant ribonuclease inhibitor (Invitrogen) in RNase-free H_2_O. cDNA was amplified by a 7500 Real-time PCR Detection System (Thermo Fisher Scientific) using a Fast SYBR Green Master Mix (Applied Biosystems, Waltham, MA, USA) containing forward and reverse primers. Target mRNA expression was normalized against *Actb* mRNA. The primers (forward and reverse) used for qPCR are as follows: *Faslg*: 5′-GCAGAAGGAACTGGCAGAAC-3′ and 5′-TTAAATGGGCCACACTCCTC-3′; *Bcl2*: 5′-GTCCTTCAGGTGTGGGATTT-3′ and GAGCAAATGCAGCCACAATAC-3′; *Bax*: 5′-AGGATGCGTCCACCAAGAAGCT-3′ and 5′-TCCGTGTCCACGTCAGCAATCA-3′; *Actb*: 5′-GGGAATGGGTCAGAAGGACT-3′ and 5′-CTTCTCCATGTCGTCCCAGT-3′.

### Pharmacokinetics of h145CSA

A mouse pharmacokinetics (PK) study was performed at the Center of Toxicology and Preclinical Sciences at the Development Center for Biotechnology (DCB), Taiwan. Male BALB/c mice (three per group) were injected intravenously with 1 mg/kg of h145CSA. Blood samples were collected at 2, 5, 15, and 30 min, 1, 2, 4, 6, 8, 24, and 48 h after injection. The amount of h145CSA remaining in plasma was quantitated by ELISA using mouse anti-human Fd antibody (clone HP6045, Sigma-Aldrich) as the capture antibody and HRP-conjugated goat anti-human kappa light chain antibody (Bethyl Laboratories) as the detecting antibody. Pharmacokinetic parameters (*t*_1/2_) were calculated by two-compartmental pharmacokinetic analysis using PKSolver 2.

### Biodistribution profile of h145CSA

The biodistribution experiment was performed at the Isotope Application Division at the Institute of Nuclear Energy Research, Taiwan. h145CSA protein was radiolabeled with Na[^131^I] using an IODO-gen (1,3,4,6-tetrachloro-3α,6α-diphenylglycouril) precoated iodination tube (Pierce, Waltham, MA, USA). Briefly, the IODO-gen tube was prewetted with Tris iodination buffer (25 mM Tris-HCl, 0.4 M NaCl, and pH 7.5). Tris iodination buffer was added, followed by Na[^131^I] in 0.1 M NaOH. After 6 min, 100 μg/40 μl of h145CSA was added to the mixture, and the mixture was left at room temperature for another 6–9 min. The sample was transferred to another tube to stop the reaction. The radiochemical purity of 96.50% ± 0.80% (mean ± standard error of the mean (SEM), *n* = 3) was determined by radio thin-layer chromatography (TLC), and the protein concentration was determined by NanoDrop (Thermo Fisher Scientific). Male BALB/c mice were injected intravenously with ^131^I-h145CSA (30 μCi, 25 μg). At 0.5, 1, 2, 6, 24, and 48 h after injection, tissues were collected and weighed and radioactivity was measured in a gamma counter (Packard Cobra II Auto-Gamma Counter, GMI, Ramsey, MN, USA). Tracer uptake was recorded as the percentage of injected dose per gram of tissue (% ID/g). The Guidelines for the Nonclinical Pharmacology/Toxicology Studies for Medicinal Product Applications, *Ministry of Health and Welfare* 1998, were followed.

### Measurements of T-cell counts *in vivo*

Spleen samples were collected from untreated control and h145F(ab’)_2_-, h145chIgGAA-, or h145CSA-treated C57BL/6 mice. Single-cell suspensions were stained with Alexa Fluor 488-conjugated anti-CD4 and PE-conjugated anti-CD8 or Alexa Fluor 488-conjugated anti-CD4 and PE-conjugated anti-FoxP3 antibodies and analyzed by flow cytometry. T-cell counts were calculated by multiplying the total spleen cell counts by the percentage of each T-cell population in the spleen. To assess CD4^+^ T cells after antibody treatment, mesenteric and pancreatic lymph nodes were collected from NOD mice on day 7 after the last injection of h145chIgGAA or h145CSA. The staining protocols to detect intracellular IFN-γ and FoxP3 in CD4^+^ T cells are as described above.

### Serum cytokine assay

Naïve C57BL/6 female mice were injected with 50 μg of h145chIgG, h145F(ab’)_2_, h145chIgGAA, or h145CSA intraperitoneally. Mice were bled at 0.5, 2, 4, 6, and 24 h after treatment and stored at −80°C until assay. The levels of TNF, IFN-γ, IL-2, and IL-6 in the serum were quantified using LEGNDplaxTM Mouse Th1 Panel following the manufacturer’s instructions (BioLegend).

### Antibody treatment of NOD mice

Efficacy studies were performed at The Jackson Laboratory and NLAC. Female NOD mice were screened for blood glucose levels twice weekly beginning at 12 weeks of age. Mice were diagnosed as diabetic when two consecutive nonfasting blood glucose levels were ≥ 250 mg/dl. At the time of diabetes onset, mice were randomized to receive intravenous or intraperitoneal injections of h145chIgGAA, h145CSA (25 μg/day), or PBS for five consecutive days. The efficacy of antibody treatment for a NOD mouse is defined as its blood glucose level dropping to and continuously maintained at ≤ 250 mg/dl from day 38 until day 84.

### Statistics

The comparisons between multiple groups were analyzed with Student’s two-tailed *t*-test and one-way or two-way ANOVA, followed by Tukey’s *post-hoc* test using GraphPad Prism version 9 software. The frequency of diabetic NOD mice responding to treatment between groups was compared by Chi-square (X2) test using GraphPad Prism version 9 software. All results are expressed as the mean ± SEM. Statistical significance was set as follows: ^*^ *p*< 0.05; ^**^ *p*< 0.01; ^***^ *p*<0.001; ^****^ *p*<0.0001.

### Ethics statement

Mouse studies were carried out in strict accordance with the recommendations in the Guide for the Care and Use of Laboratory Animals (published by The Chinese-Taipei Society of Laboratory Animal Sciences, 2007 or the National Research Council, 2011). All animal procedures and experimental protocols were approved by the Institutional Animal Care and Use Committee (IACUC) of the following institutes: Industrial Technology Research Institute (permit: ITRI-IACUC-2010-050M, ITRI-IACUC-2019-070, ITRI-IACUC-2019-071, ITRI-IACUC-2021-043); The Jackson Laboratory (JAX-13060); National Laboratory Animal Center, National Applied Research Laboratories, Taiwan (NLAC(TN)-110-M-007, NLAC(TN)-111-M-001, NLAC-111-M-022); Center of Toxicology and Preclinical Sciences at the Development Center for Biotechnology, Taiwan (DCB-2010-TP-001); and Institute of Nuclear Energy Research, Taiwan (No. 99064).

## Results

### Generation and characterization of anti-CD3 Collabody and its bivalent antibody counterparts

To generate a trivalent anti-mouse CD3 Collabody in F(ab’)_3_ format, we used a self-trimerizing collagen-like peptide (30) and adopted a hamster/human chimeric Fab version of hamster anti-mouse CD3ε monoclonal antibody (clone 145-2C11) instead of hamster/mouse chimeric Fab for the sake of expression efficiency. The heavy chain Fd region plus the hinge region were fused with a C-terminal collagen-like peptide (GPP)_10_ and co-expressed with the kappa light chain in CHO-S cells. A recombinant hamster/human chimeric 145-collagen scaffold-antibody (h145CSA) was thus generated. Two mouse chimeric IgG antibodies were generated by fusion of the same hamster/human chimeric Fab sequence to the Fc region of mouse IgG_2a_ with or without the two leucine to alanine substitutions at amino acids 234 and 235, and they were designated h145chIgGAA and h145chIgG, respectively. h145F(ab’)_2_, an FcR nonbinding control, was prepared by pepsin digestion of h145hIgG_1_. The structure features of these anti-CD3 antibody variants are shown (**Figure 1A**; **Table 1**). The molecular mass and structure integrity of purified anti-CD3 antibodies were analyzed by SDS-PAGE and size exclusion chromatography (SEC)-HPLC (**Supplementary Figures S1A, B**), respectively. The results confirmed that these anti-CD3 antibody variants were expressed with the expected molecular mass and homogeneity without any higher-order structures that can contribute to unwanted multivalent binding effects.

**Table 1:** Anti-CD3 antibodies used in this study.

Name	Type	*V_H_*/*V_L_* origin	CH_1_/C_L_ sequence	Fc format	Binding to FcR
145-2C11	Hamster mAb	145-2C11	Hamster IgG_1_, hamster C_L_κ	Hamster IgG_1_	+
h145F(ab’)_2_	Chimeric F(ab’)_2_	145-2C11	Human CH_1_, human C_L_κ	None	−
h145chIgG	Chimeric mAb	145-2C11	Human CH_1_, human C_L_κ	Mouse IgG_2a_	+
h145chIgGAA	Chimeric mAb	145-2C11	Human CH_1_, human C_L_κ	Mouse IgG_2a_ (mutation at aa. 234, 235, Leu to Ala)	Reduced
h145CSA	Chimeric F(ab’)_3_	145-2C11	Human CH_1_, human C_L_κ	None (substituted by (GPP)_10_)	−

FcR, Fc receptor; CH_1_, first constant Ig domain of the heavy chain; C_L_, light chain constant region.

### Anti-CD3 CSA is superior to bivalent antibodies in binding strength and ability to downregulate cell surface TCR

h145CSA and anti-CD3 antibody variants, h145F(ab’)_2_ and h145chIgGAA, were compared to the parental 145-2C11 for their direct binding potencies to CD3 on T cells. The equilibrium binding potencies of all three anti-CD3 antibody variants were in the nanomolar range, similar to the parental 145-2C11, indicating that the binding capability of the Fv portion of anti-CD3 antibodies to CD3 on the T-cell surface is comparable to 145-2C11 (**Figure 1B**). The binding strength of trivalent h145CSA was about threefold that of h145chIgGAA (0.95 and 2.51 nM, respectively). The experiment was performed to evaluate the effect of antibody valency (tri- vs. bivalency) on its dissociation rate. Mouse T cells were preincubated with different concentrations of h145CSA and anti-CD3 antibody variants on ice, followed by adding a subsaturating concentration of Alexa Fluor 647-conjugated 145-2C11. Results showed that all three anti-CD3 antibodies competed against the binding of fluorescent-dye conjugated 145-2C11 to mouse T cells in a dose-dependent manner (**Figure 1C**). Comparing their IC_50_ values, it is evident that it took only one-third the concentration of h145CSA (IC_50 =_ 0.78 nM) to achieve the same inhibitory effect as that for h145chIgAA (IC_50 =_ 2.2 nM) (**Figure 1C**). Therefore, trivalent h145CSA binds to the TCR/CD3 complex on T cells with greater binding strength than bivalent h145chIgAA, thus resulting in a slower *k*_off_ rate. The anti-CD3 antibody variants were further examined for their ability to downregulate TCR/CD3 complex on the cell surface at 37°C. h145CSA was more efficient than its bivalent h145chIgAA, h145F(ab’)_2_, and its parental 145-2C11 in reducing TCR/CD3 on purified total T cells as well as CD4^+^ and CD8^+^ T cells (**Figure 1D**; **Supplementary Figure S2**). At antibody concentrations from 0.6 to 6 nM (*p*< 0.0001, h145CSA compared with other bivalent antibodies at different antibody concentrations at 0.6 nM; *p*< 0.0001, h145CSA compared to h145F(ab’)_2_ and h145chIgGAA at 6 nM; *p*< 0.05, h145CSA compared to h145F(ab’)_2_ at 60 nM) (**Figure 1D**). These data demonstrate that trivalent anti-CD3 CSA has a greater ability to downregulate cell surface TCR/CD3 complex than its bivalent variants.

### Naïve and activated CD4^+^ T cells differ in their susceptibility to trivalent anti-CD3 CSA- and bivalent anti-CD3 IgG-induced apoptosis

**Figure 2:**
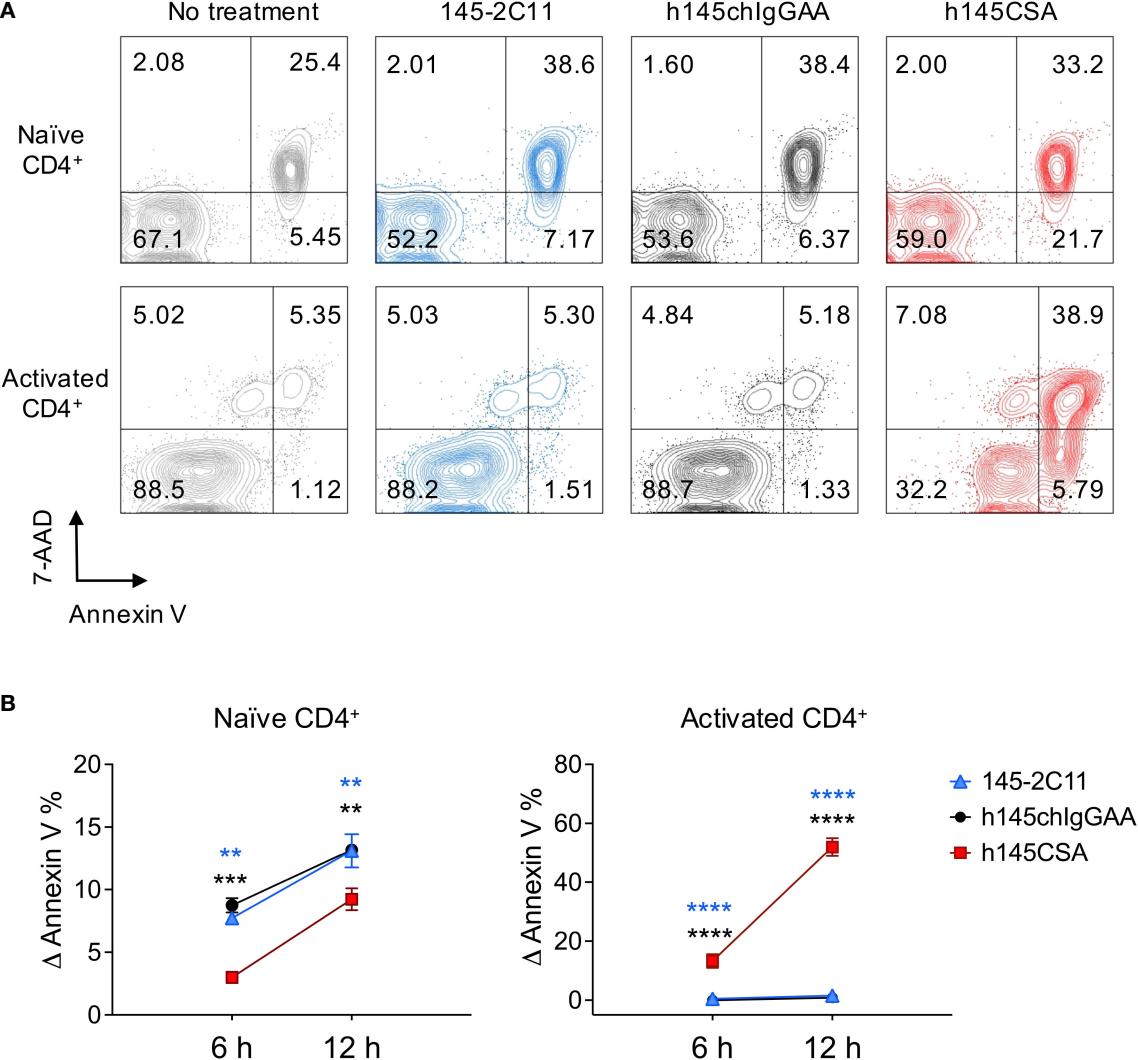
h145CSA is more efficient than bivalent anti-CD3 antibodies in inducing activated but not naïve CD4^+^ T-cell apoptosis. **(A, B)** Spleens from healthy female C57BL/6 mice at 6–12 weeks of age were collected. Naïve splenic CD4^+^ T cells were enriched by using Mouse Naïve CD4 T-Cell Isolation Kit. **(A)** Freshly isolated naïve and preactivated CD4^+^ T cells were treated with 10 μg/ml of 145-2C11, h145chIgGAA, or h145CSA for 12 h before staining with FITC-conjugated Annexin V and 7-AAD. Cells were subject to flow cytometric analysis. One representative of three independent experiments is presented. **(B)** Freshly isolated naïve and preactivated CD4^+^ T cells were treated with 145-2C11, h145chIgGAA, or h145CSA anti-CD3 antibody for 6 and 12h. Percent-specific apoptotic cells were calculated as Δ Annexin V % = % Annexin V^+^ cells with anti-CD3 antibody treatment – % Annexin V^+^ cells in medium alone. Data were pooled from three (naïve) or four (preactivated) independent experiments. The bars represent the mean ± SEM. Data were analyzed by two-way ANOVA followed by Tukey’s *post-hoc* test. ^**^ *p*< 0.01; ^***^ *p*< 0.001; ^****^ *p*< 0.0001.

Induction of T-cell apoptosis is a way to achieve immune tolerance. Trivalent h145CSA was compared to bivalent 145-2C11 and h145chIgGAA for its ability to induce apoptosis in naïve and activated CD4^+^ T cells. Treatment with either h145CSA or bivalent anti-CD3 antibodies induced naïve CD4^+^ T-cell apoptosis as early as 6 h and continued until 12 h after stimulation, when 7%–13% of cells were apoptotic. However, h145CSA-induced naïve CD4^+^ T-cell death was significantly lower than bivalent antibodies at 6 and 12 h after treatment (**Figures 2A, B**). Interestingly, only trivalent h145CSA, but not the bivalent antibodies, induced activated CD4^+^ T-cell death (**Figures 2A, B**). As many as about 50% of trivalent h145CSA-treated cells were apoptotic at 12 h after treatment. These results indicate that activated CD4^+^ T cells are more susceptible than naïve cells to anti-CD3 CSA-induced cell death and that anti-CD3 CSA but not anti-CD3 IgG antibodies is effective in inducing activated CD4^+^ T-cell apoptosis.

### Th1 cells are more susceptible than Treg cells to anti-CD3 CSA-induced apoptosis

**Figure 3:**
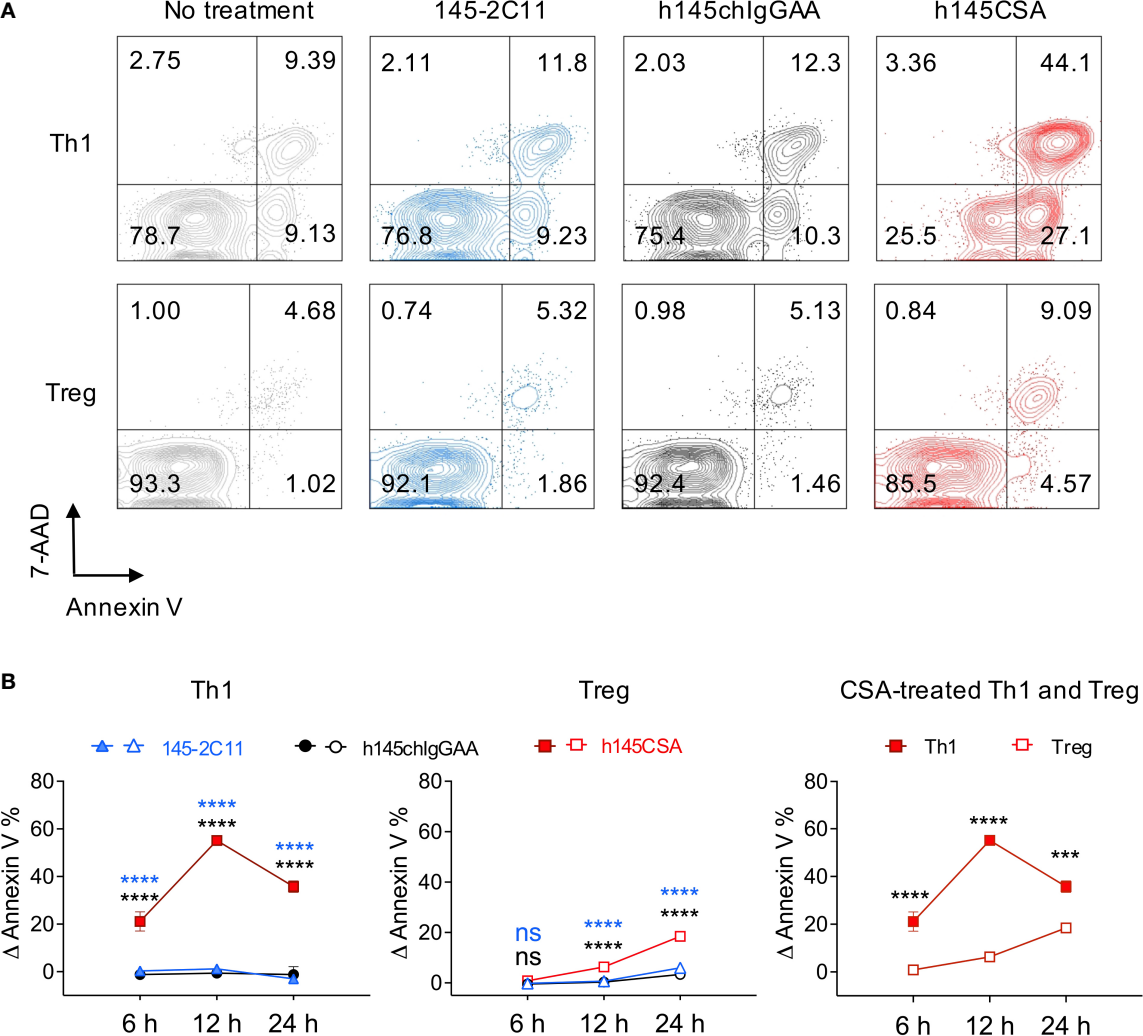
Th1 cells are more susceptible than Treg cells to h145CSA-induced apoptosis. **(A, B)** Spleens from healthy female C57BL/6 mice at 6–12 weeks of age were collected. Naïve splenic CD4^+^ T cells were enriched by using Mouse Naïve CD4 T-Cell Isolation Kit and subjected to Th1 (anti-CD3 Ab, anti-CD28 Ab, IL-2, IL-12, and anti-IL-4 Ab) and Treg (anti-CD3 Ab, anti-CD28 Ab, IL-2, TGF-β, anti-IL-4 Ab, and anti-IFN-γ Ab) skewing conditions, respectively. Th1 and Treg cells were exposed to 10 µg/ml of 145-2C11, h145chIgGAA, h145CSA, or medium alone for 12 h **(A)** or 6, 12, and 24 h **(B)** before staining with FITC-conjugated Annexin V and 7-AAD. Cells were subjected to flow cytometric analysis. **(A)** Numbers on the upper right corner of contour plots show Annexin V^+^7-AAD^+^ apoptotic cells. One representative of three independent experiments is presented. **(B)** Percent-specific apoptotic cells were calculated as Δ Annexin V % = % Annexin V^+^ cells with anti-CD3 antibody treatment – % Annexin V^+^ cells in medium alone. Data were pooled from three independent experiments. The bars represent the mean ± SEM. Data were analyzed by two-way ANOVA followed by Tukey’s (Th1 and Treg) or Bonferroni’s (h145CSA treated Th1 and Treg) *post-hoc* test. ^***^ *p*< 0.001; ^****^ *p*< 0.0001; ns, not significant.

CD4^+^ T cells were skewed to Th1 (CD4^+^IFN-γ^+^ cells, 85.9% of total CD4^+^ cells) and Treg (CD4^+^FoxP3^+^ cells, 85.1% of total CD4^+^ cells) (**Supplementary Figure S3**) and treated with trivalent h145CSA, bivalent 145-2C11, or h145chIgGAA antibody. Treatment with h145CSA induced significantly higher percentages of Th1 and Treg cell death compared to 145-2C11 and h145IgGAA at all three time points tested except for Treg at 6 h (**Figures 3A, B**). However, the kinetics and susceptibility of Th1 and Treg cells to h145CSA were much different (**Figure 3B**, right panel). h145CSA-induced Th1 cell apoptosis occurred as early as 6 h (Δ Annexin V^+^ cells: 21.1% ± 4.1%), and the percentage of apoptotic cells reached 55.1% ± 1.28% at 12 h after stimulation. Percent h145CSA-induced Th1 cell apoptosis remained high at 24 h (35.8% ± 2.4%) but lower than that at the 12-h time point due to an increase of cell death in control (**Supplementary Figure S4**). h145CSA-induced Treg cell death was significantly lower than Th1 cells at all three time points and the percentage of cell death remained as low as 18.5% ± 0.7% at 24 h after stimulation (Δ Annexin V^+^ cells: 0.84% ± 1.1% at 6 h, 6.3% ± 0.7% at 12 h, and 18.5% ± 0.7% at 24 h) (**Figure 3B**). These results indicate that anti-CD3 CSA is more effective than bivalent anti-CD3 IgG antibodies in inducing Th1 and Treg cell death and that Th1 cells are more susceptible than Treg cells to anti-CD3 CSA-induced apoptosis.

### Anti-CD3 CSA induces Th1 cell apoptosis by upregulating FasL expression

**Figure 4:**
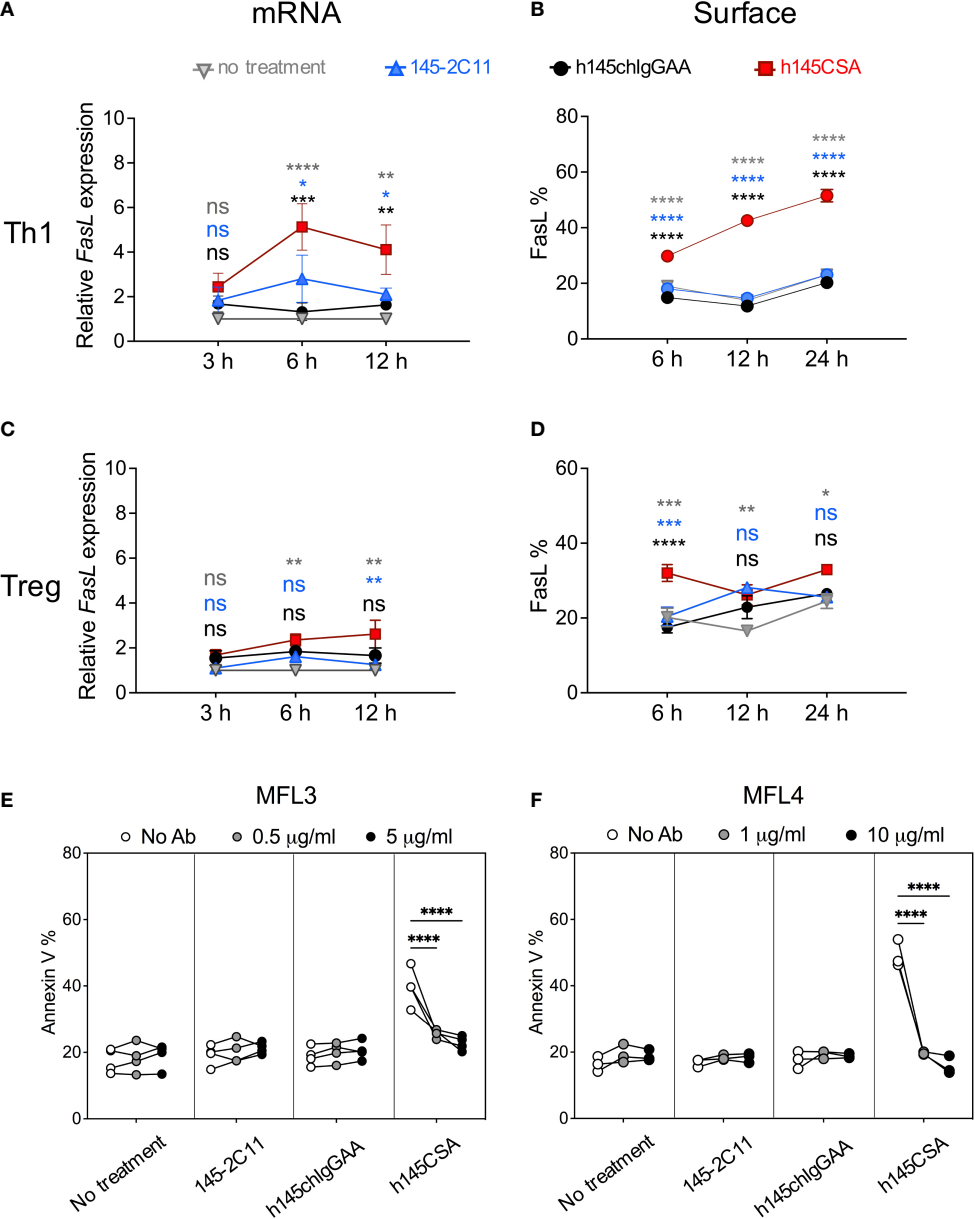
h145CSA induces Th1 cell apoptosis by upregulating FasL expression. Spleens from healthy female C57BL/6 mice at 6–12 weeks of age were collected. Naïve splenic CD4^+^ T cells were enriched by using Mouse Naïve CD4 T-Cell Isolation Kit and subjected to Th1 and Treg skewing conditions, respectively. *In vitro*-differentiated Th1 **(A, B, E, F)** and Treg **(C, D)** cells were treated with 10 µg/ml of 145-2C11, h145chIgGAA, h145CSA or cultured in medium alone. **(A, C)** Antibody-treated or antibody-untreated Th1 **(A)** or Treg **(C)** cells were subjected to RNA extraction 3, 6, and 12 h after culture. *Fasl* mRNA expression was analyzed by RT-qPCR and normalized against *Actb* mRNA. The relative expression level of *Fasl* was calculated as the normalized *Fasl* mRNA expression of cells with antibody treatment divided by normalized *Fasl* mRNA expression of cells in medium alone at the same time point. **(B, D)** Cells were cultured for 6, 12, and 24 h before staining with biotin-conjugated anti-FasL antibody, followed by APC-conjugated streptavidin and subjected to flow cytometric analysis. The percentage of total cells expressing FasL on the cell surface are shown. **(E, F)** Cells were treated with 10 μg/ml of the indicated anti-CD3 antibody and unconjugated anti-FasL antibody from either MFL3 **(E)** or MFL4 **(F)** clones for 12 h and stained with FITC-conjugated Annexin V. The percentage of cells expressing Annexin V is shown. Data were pooled from three **(C, F)** or four **(A, B, D, E)** independent experiments. The bars represent the mean ± SEM. Data were analyzed by two-way ANOVA followed by Tukey’s *post-hoc* test. ^*^ *p*< 0.05; ^**^ *p*< 0.01; ^***^ *p<* 0.001; ^****^ *p*< 0.0001; ns, not significant.

Fas/FasL interaction and mitochondrial cell death effector molecules have been demonstrated to regulate activated T-cell death (31–34). h145CSA, h145IgGAA, and 145-2C11 were compared for their ability to induce Fas/FasL, Bcl-2, and Bax in Th1 cells. Results showed that treatment with any of the three antibodies did not change the level of Fas on Th1 cell surface (**Supplementary Figure S5A**). Neither was there any change in *Bcl-2* and *Bax* at the transcription level after treatment with bivalent or trivalent anti-CD3 antibodies at either 3 or 6 h after treatment (**Supplementary Figures S5B, C**). However, h145CSA treatment significantly increased FasL at both the transcript (6 and 12 h) and protein (6, 12, 24 h) levels in Th1 cells as compared to the bivalent anti-CD3 antibodies (**Figures 4A, B**; **Supplementary Figure S6A**). In contrast, stimulation with h145CSA only slightly increased the expression of FasL on Treg cells (**Figures 4C, D**; **Supplementary Figure S6B**) when compared to the bivalent antibodies. Importantly, blocking FasL by antibodies from either MFL3 (**Figure 4E**) or MFL4 clone (**Figure 4F**) completely reversed h145CSA-induced Th1 cell apoptosis. These data together indicate that anti-CD3 CSA induces higher FasL expression in Th1 cells than in Treg cells and that anti-CD3 CSA triggers Th1 cell death by upregulating FasL.

### Pharmacokinetics and its biodistribution profile of anti-CD3 CSA

The structure and molecular mass of a protein affect its pharmacokinetics and tissue penetration profile, and these properties further affect the dosing regimen and therapeutic potency of the antibody *in vivo* (35, 36). We conducted a pharmacokinetic study of h145CSA in BALB/c mice. Mice were injected intravenously with a single dose of h145CSA at 1 mg/kg of body weight. Due to the lack of the Fc domain of h145CSA, the PK profile revealed a rapid increase followed by a decrease of the antibody concentration in the circulation after injection (**Supplementary Figure S7A**). The terminal half-life of h145CSA is approximately 11.2 h, similar to that of the F(ab’)2 fragment but much shorter than IgG, which is protected by neonatal Fc receptor from intracellular degradation (37–39). To evaluate tissue penetration of h145CSA, BALB/c mice were intravenously administered ^131^I-labeled h145CSA. Selected organs were collected, and their radioactivity was determined. By total radioactivity count, blood, bone, spleen, and small intestine had significantly higher counts than the brain from 0.5 to 48 h after injection (**Supplementary Figure S7B**). By tissue-to-blood ratio, not only bone, spleen, and small intestine but also the thymus had higher radioactivity per gram than the brain at 48 h after injection (**Supplementary Figure S7C**). It appears that anti-CD3 CSA does not cross the blood–brain barrier and yet successfully penetrates other tissues, including lymphoid tissues, to interact with T cells.

### Multiple dosing of anti-CD3 CSA efficiently reduces splenic T cells followed by rapid recovery kinetics in naïve mice

**Figure 5:**
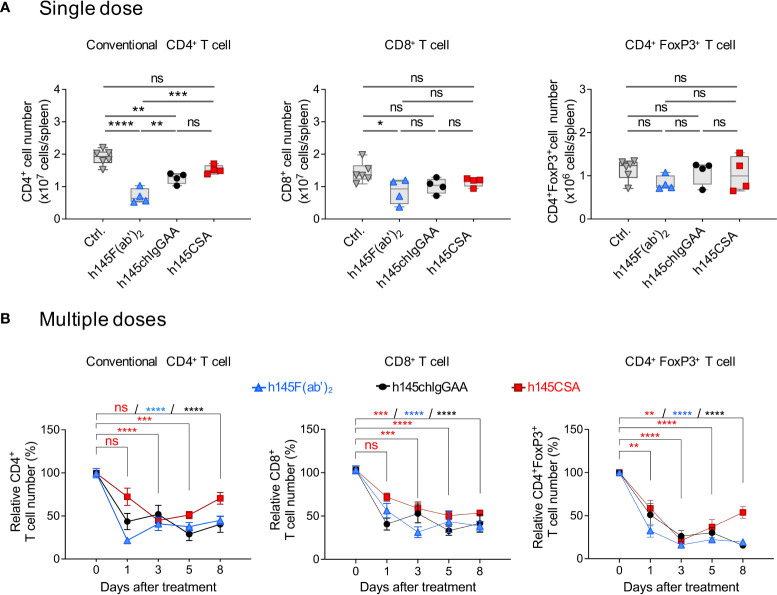
Multiple doses of h145CSA efficiently deplete splenic T cells in naïve mice. Female naïve C57BL/6 mice at 6–12 weeks of age were intraperitoneally injected with a single dose (50 μg) **(A)** or multiple doses (25 μg/day for five consecutive days) **(B)** of h145F(ab**’**)_2_, h145chIgGAA, h145CSA, or PBS (ctrl). Spleens were collected at the indicated time points after injection. Spleen cells were stained with either Alexa Fluor 488-conjugated anti-CD4/PE-conjugated anti-CD8 or with Alexa Fluor 488-conjugated anti-CD4/PE-conjugated anti-FoxP3 antibodies, followed by flow cytometric analysis. **(A)** The absolute numbers of conventional CD4^+^ (left panel), CD8^+^ (middle panel), and Treg (right panel) cells from mice receiving a single dose of anti-CD3 antibodies were counted on day 1 after injection. (ctrl., *n* = 6; h145F(ab**’**)_2_, h145chIgGAA, h145CSA, *n* = 4 each). **(B)** The cell numbers of conventional CD4^+^ (left panel), CD8^+^ (middle panel), and Treg (right panel) cells from mice receiving five doses of anti-CD3 antibodies were counted on days 1, 3, 5, and 8 after the last injection. The relative cell number was calculated as the absolute cell number divided by the average cell number of control mice. (ctrl., *n* = 5–6; h145F(ab)_2_, *n* = 3–6; h145chIgGAA, *n* = 3–6; h145CSA, *n* = 4–6). Data were analyzed by one-way **(A)** or two-way **(B)** ANOVA followed by Tukey**’**s *post-hoc* test. ^*^ *p*< 0.05; ^**^ *p*< 0.01; ^***^ *p*< 0.001; ^****^ *p*< 0.0001; ns, not significant.

Dose titration of h145CSA ranging from 0.03 to 50 μg showed that administrating 50 μg of h145CSA resulted in a reduction of CD4^+^ and CD8^+^ T cells in naïve mice within 1 h after injection (**Supplementary Figures S8A, B**). An early study show that a single dose of 145-C11 at 400 μg depleted 40% of CD4^+^ T cells in naïve mice (12). h145CSA, h145F(ab’)_2_, and h145chIgGAA were compared for their efficiency in depleting different T-cell populations in naïve mice. While h145F(ab’)_2_ and h145chIgGAA treatments at 50 μg significantly reduced the numbers of splenic conventional CD4^+^ T cells on day 1 after administration, h145CSA treatment did not (**Figure 5A**). With the exception of h145F(ab’)_2_ for CD8^+^ T cells, a single dose of either h145chIgGAA or h145CSA was not sufficient to deplete splenic CD8^+^ T cells and CD4^+^Foxp3^+^Treg cells. Thus, we adjusted the dosing regimen. Naïve mice were then given five consecutive daily injections of 25 μg of h145CSA, h145F(ab’)_2_, or h145chIgGAA. Those mice that received five doses of h145CSA, h145F(ab’)_2_, or h145chIgGAA significantly reduced splenic conventional CD4^+^, CD8^+^ T cells, and CD4^+^Foxp3^+^ Treg cells by day 1 (**Figure 5B**). Their efficiency varied, with h145CSA as the least on day 1 but comparable to others by day 3 (**Figure 5B**). While CD8^+^ T cells and CD4^+^Foxp3^+^ Treg cells in h145CSA-, h145F(ab’)_2_-, and h145chIgGAA-treated mice remained significantly lower than control mice (day 0) and lasted beyond day 8 after the last treatment, h145CSA treatment allowed earlier recovery of conventional CD4^+^ T cells (within 8 days after last treatment) than its bivalent counterparts (beyond day 8) (**Figure 5B**). Anti-CD3 CSA with such characteristics is further tested for its mitogenicity in mice.

### Anti-CD3 CSA induces a weak mitogenic response *in vivo*

**Figure 6:**
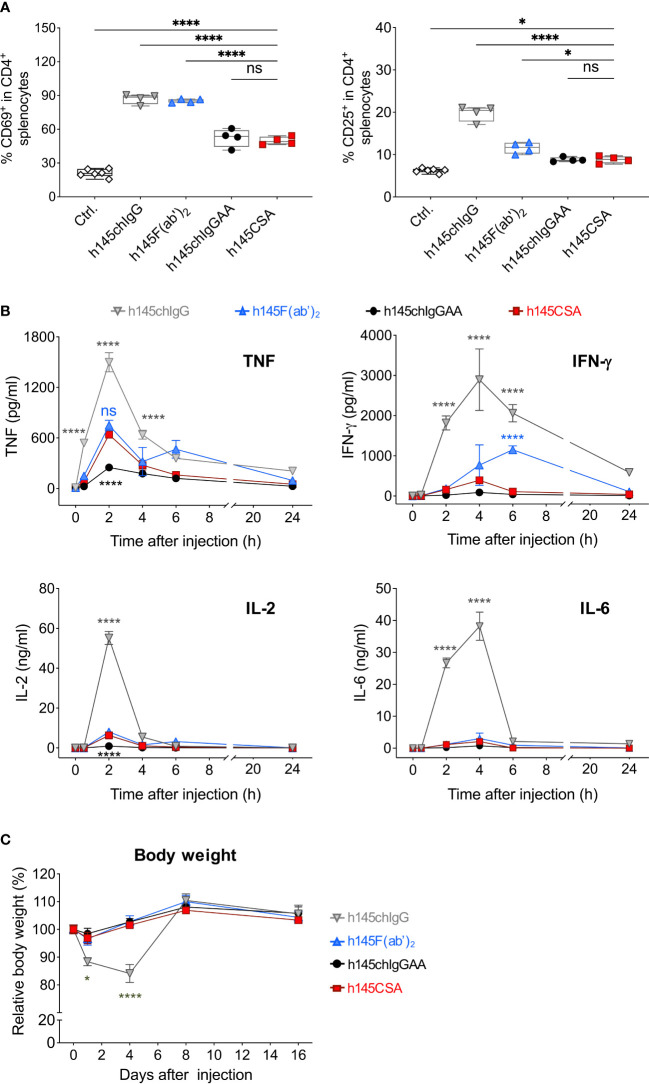
Comparing the mitogenic response of mice after receiving different anti-CD3 antibodies. Female naïve C57BL/6 mice at 6–12 weeks of age were intraperitoneally injected with 50 µg of h145chIgG, h145F(ab’)_2_, h145chIgGAA, h145CSA, or PBS (ctrl.). **(A)** Spleens were collected on day 1 after injection. Spleen cells were stained with Alexa Fluor 488-conjugated anti-CD4, PE-conjugated anti-CD69, and APC-conjugated anti-CD25 antibodies, followed by flow cytometric analysis. Percent CD69^+^ or CD25^+^ in splenic CD4**^+^** T-cell population are shown. Each symbol represents the datum of one mouse. (ctrl., *n* = 6; h145chIgG, h145F(ab’)_2_, h145chIgGAA, h145CSA, *n* = 4 each). **(B)** Serum was collected from mice 0, 2, 4, 6, and 24 h after injection. The levels of TNF, IFN-γ, IL-2, and IL-6 were quantified by multiplex analysis. Each datum point is the pool of serum from three to four mice. Cytokine levels in untreated mice are as follows: TNF, 7.15–22.9 pg/ml; IFN-γ, 0.75–4.19 pg/ml; IL-2, 0.7–1.2 pg/ml; and IL-6, 0.38–1.14 pg/ml. **(C)** The body weight of the mice in each group was measured on days 0, 1, 4, 8, and 16 days after injection. Four mice were included in each treatment group. In **(B**, **C)**, data in each treatment group were compared to mice receiving h145CSA. The bars represent the mean ± SEM. The data were analyzed by a two-way ANOVA followed by the Tukey’s *post-hoc* test. ^*^ *p*< 0.05; ^****^ *p*< 0.0001; ns, not significant.

It is reported that acute morbidity caused by anti-CD3 antibodies is due primarily to T-cell activation and cytokine release as a result of antibody-mediated crosslinking of CD3 on T cells and FcγRs on accessory cells (10). To compare the mitogenicity of h145CSA with other antibodies, mice were intraperitoneally given 50 µg of h145CSA, h145chIgG, h145F(ab’)_2_, or h145chIgGAA. Splenic CD4^+^ T-cell expressions of CD69 and CD25 and cytokine production were analyzed. While administration of each antibody significantly increased CD69 and CD25 expressions on splenic CD4^+^ T cells, the increase of CD69 in h145CSA- and h145chIgGAA-treated mice were only half of those treated with h145chIgG and h145F(ab’)_2_ (**Figure 6A**). Moreover, h145chIgG induced high levels of TNF, IFN-γ, IL-2, and IL-6 in the blood stream within the first 24 h after administration. While h145chIgGAA induced minimum-to-none levels of cytokines, h145CSA treatment induced low-to-minimum levels of all cytokines tested except for TNF, which was at a level comparable to that by h145F(ab’)_2_ (**Figure 6B**). Body weight loss, a consequence of cytokine release, was observed in h145chIgG-treated mice but not in mice receiving h145CSA, h145F(ab’)_2_, and h145chIgGAA (**Figure 6C**). Thus, it appears that h145CSA possesses the characteristics that favor *in vivo* use.

### Anti-CD3 CSA is therapeutically more efficacious than anti-CD3 IgGAA in treating recent-onset diabetic NOD mice

**Figure 7:**
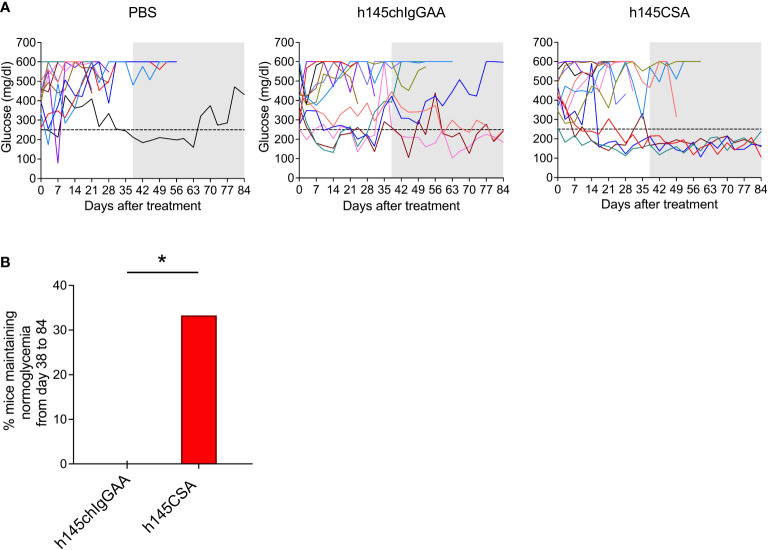
Comparison of the therapeutic effects of h145chIgGAA and h145CSA. Female NOD mice were randomized at the time of diabetes onset (two consecutive nonfasting blood glucose levels ≥ 250 mg/dl) to receive five consecutive intravenous injections of 25 μg/day of h145chIgGAA, h145CSA, or PBS (*n* = 12 in each group). **(A)** Glucose levels of PBS-injected, h145chIgGAA-treated, and h145CSA-treated mice are shown. Each line in different color represents the glucose level of each individual mouse from the day of treatment (day 0) until day 84. **(B)** The bar graph shows the percentage of h145chIgGAA- or h145CSA-treated mice maintaining normoglycemia from days 38 to 84. The frequency of the response to treatment between groups was compared by a Chi-square (*χ*²) test. ^*^ *p*< 0.05.

h145CSA was compared to h145chIgGAA for its therapeutic potency in recent-onset female diabetic NOD mice. With two consecutive blood glucose level readings higher than 250 mg/dl, mice were randomized to receive intravenous injections of either h145CSA or h145chIgGAA at 25 μg/day for five consecutive days. The glucose levels and body weight of individual mice were followed. Results showed that some diabetic mice began to respond to h145CSA as early as 3 to 17 days after the start of treatment, and these responsive mice remained stably normoglycemic until day 84 (**Figure 7A**). Blood glucose levels in mice treated with h145chIgGAA fluctuated. At some time points, it may drop below 250 mg/dl but cannot be maintained stably (**Figure 7A**). Between days 38 and 84 after the start of treatment, four out of 12 diabetic NOD mice treated with h145CSA remained normoglycemic, whereas none of the h145chIgGAA-treated mice did (**Figure 7B**). The body weight of the cured mice was 25.3, 25.0, 27.0, and 29.4 g by day 84 after treatment. They are within the range of the weights of healthy female NOD mice at 24 (25.9 ± 1.7 g) to 30 weeks (27.2 ± 2.1 g) of age as published by Jackson Labs (https://www.jax.org/jax-mice-and-services/strain-data-sheet-pages/body-weight-chart-001976). These results indicate that anti-CD3 CSA can maintain blood glucose levels in mice responding to the treatment. Although its cure rate is 33%, it is superior to anti-CD3 IgGAA in treating recent-onset diabetes in NOD mice.

### The efficacy of anti-CD3 CSA correlates with the reduction of CD4^+^IFN-γ^+^ T cells

**Figure 8:**
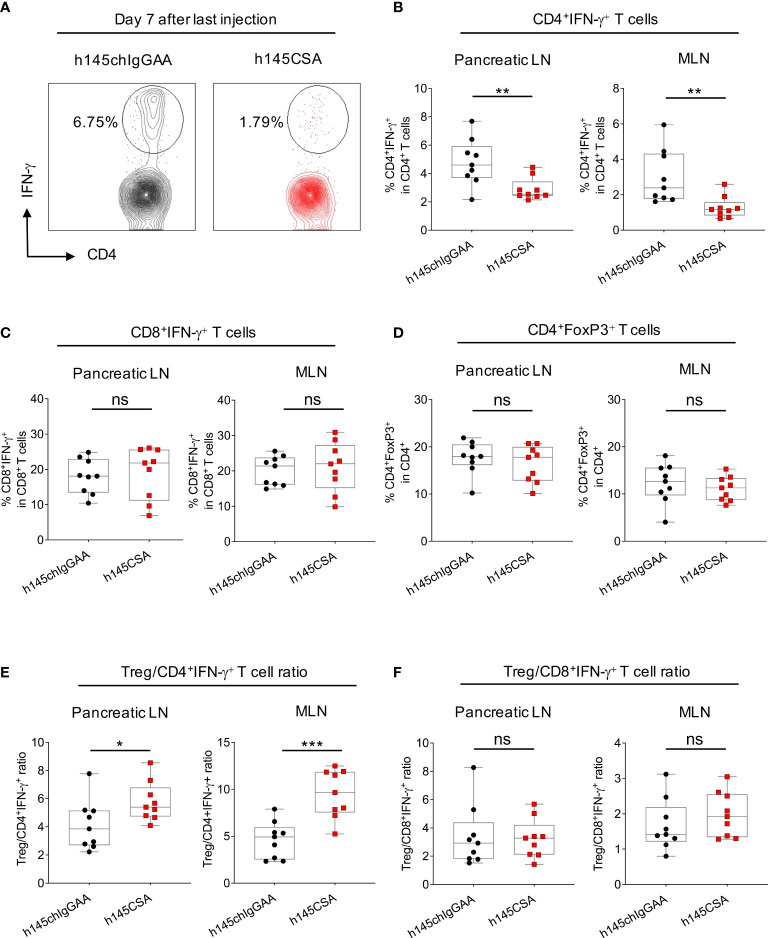
Comparing h145CSA treatment to h145chIgGAA for effectiveness in reducing Th1 cells and Treg cells in lymph nodes. Female NOD mice were randomized at the time of diabetes onset (two consecutive nonfasting blood glucose levels ≥ 250 mg/dl) to receive five consecutive intraperitoneal injections of 25 μg/day of h145chIgGAA or h145CSA (*n* = 9 each). Pancreatic and mesenteric lymph node cells were obtained from both groups of mice on day 7 after the last of five consecutive intraperitoneal injections. **(A, B)** Cells were stained with Alexa Fluor 488-conjugated anti-CD4 and Alexa Fluor 647-conjugated anti-IFNγ antibodies and **(C)** with PerCP/Cy5.5-conjugated anti-CD8 and Alexa Fluor 647-conjugated anti-IFNγ antibodies and **(D)** with Alexa Fluor 488-conjugated anti-CD4 and PE-conjugated anti-FoxP3 antibodies. Cells were subjected to flow cytometric analysis. **(A)** Representative plots showing the percentage of IFN-γ^+^ cells in CD4 gate of pancreatic lymph node (Pancreatic LN) cells obtained from h145chIgGAA- and h145CSA-treated mice. The percentage of IFN-γ^+^ cells in the CD4^+^ **(B)** and CD8^+^ **(C)** T-cells and FoxP3^+^-cell population in CD4^+^ T cells **(D)** in pancreatic (Pancreatic LN) and mesenteric (MLN) lymph nodes. Treg to Th1 **(E)** and CD8^+^IFN-γ^+^ T-cell **(F)** ratio in h145chIgGAA- and h145CSA-treated groups. Each symbol represents a datum from one mouse. Data were analyzed by Student’s *t*-test. ^*^ *p*< 0.05; ^**^ *p*< 0.01; ^***^ *p*< 0.001; ns, not significant.

Compared to h145chIgGAA treatment, mice receiving h145CSA had a significantly lower percentage of CD4^+^IFN-γ^+^ T cells in pancreatic and mesenteric lymph nodes on day 7 after the last treatment (**Figures 8A, B**). While both the percentages of CD8^+^IFN-γ^+^ T cells (**Figure 8C**) and Treg (**Figure 8D**) as well as Treg/CD8^+^IFN-γ^+^ T cell ratio (**Figure 8F**) in mice with either treatment were comparable, h145CSA treatment resulted in higher Treg/Th1 ratio (**Figure 8E**) than h145chIgGAA treatment. These results indicate that anti-CD3CSA is therapeutically efficacious and that the efficacy is associated with its ability to reduce Th1 cells and increase Treg/Th1 ratio.

## Discussion

In this study, we characterized an anti-CD3 recombinant h145 collagen scaffold antibody and compared it to its bivalent counterparts. *In vitro*, studies showed that h145CSA is superior to the bivalent ones in inducing Th1 cell apoptosis. Multiple doses of h145CSA are sufficient to deplete T cells in mice. Multiple injections of h145CSA skew one-third of recent-onset diabetic mice back to normoglycemia, and the effect is long-lasting. The efficacy of h145CSA is accompanied by a significant reduction of Th1 cells and reversion of the Th1/Treg ratio in pancreatic as well as mesenteric lymph nodes. This is the first study to show that CSA has therapeutic efficacy in an autoimmune setting.

Collagen scaffold antibody, one form of Collabodies, is a trivalent antibody with its Fc portions replaced by a triplex collagen-like peptide scaffold. Our previous studies demonstrated that an anti-CD3 scFv (single-chain Fv) N-terminus fused to a collagen-like peptide scaffold (Gly-Pro-Pro)_10_ is capable of forming a trivalent antibody (30). However, we discovered that the level of trivalent scFv protein expression is low (personal observation). Another study employing the chimeric anti-CD20 scFv-Fc fusion protein by fusing scFv with human IgG_1_ hinge and Fc regions showed that the fusion protein in its purified form contains multimeric as well as monomeric components (40). The fusion protein multimers through domain exchange and crosspairing of the variable domain results in the formation of heterogeneous antibody variants (40). Hence, instead of scFv, we used Fab fragment as an antigen-binding domain. By using Fab, antibody trimerization is driven by triplex-forming collage-like peptide and avoids multimerization through the variable domain. With the modification, we were able to generate recombinant h145CSA as a homogenous trimer. It is of interest to note that the fusion construct of hamster/mouse chimeric Fab could not be expressed as a soluble protein through the CHO-S cell expression system. By adopting a hamster/human chimeric Fab sequence, a trivalent anti-mouse CD3 collagen scaffold antibody (h145CSA) was successfully produced as a soluble protein. The soluble protein could be purified as a homotrimer by affinity and size-exclusion chromatography to high purity, making it suitable for use as a therapeutic.

CD3-specific antibodies binding to the CD3/TCR complex induce internalization or shedding of the complex (2). It has been shown that multivalent crosslinking of CD3/TCR by CD3-specific multimeric IgM or by IgG through the Fc portion and Fc receptor interaction is an efficient way to downregulate surface TCRs (14, 41). Reducing cell surface CD3/TCR puts a stop to signal transduction and prevents T cells from hyperactivation (42). It is reported that visilizumab (HuM291-IgG2M3, an FcR reduced-binding humanized anti-CD3 antibody) unlike other FcR reduced-binding antibodies targeting CD3, fails to downregulate TCR on cell surface. As a result, ERK2 phosphorylation in activated human T cells is sustained and is clinically intolerable (4, 28). We showed that trivalent anti-CD3 CSA is more efficient than its bivalent counterparts in inducing TCR reduction, ensuing T-cell unresponsiveness to antigen stimulation. Thus, it appears that anti-CD3 CSA treatment is able to downregulate cell surface CD3/TCR complex, thus preventing T-cell hyperactivation, a feature that is much desired for clinical use.

The induction of apoptosis is an important mechanism of immune tolerance induced by anti-CD3 antibodies. Through its F(ab’)_2_ portion, soluble anti-CD3 antibodies can transduce TCR signals and promote T-cell activation-induced cell death (AICD) *in vitro* (3, 4, 43). Soluble anti-CD3 antibodies have been reported to cause apoptosis of antigen-activated murine T cells and immobilize anti-CD3 antibody-activated human peripheral T cells (3, 4). Interestingly, AICD induced by anti-CD3 antibodies is selective for conventional T cells, especially Th1 cells, with limited effects on CD4^+^FoxP3^+^ Treg (5). We showed in this study that h145CSA is superior to its bivalent counterparts in inducing Th1 cell apoptosis through upregulation of FasL expression. We tested h145CSA and bivalent antibodies for their ability to induce apoptosis at a broad range of concentrations, from 0.1 and 1.0 to 10 μg/ml (**Supplementary Figure S9**), and found that h145CSA at all concentrations tested induced significantly higher percentages of Th1 cell death than others at the same concentration. Induction of apoptosis by TCR signaling requires activation of ERK2 to induce FasL expression (44). Crosslinking of FcR reduced-binding anti-CD3 antibody by anti-IgG restores TCR downstream ERK activation in Th1 cell clones (45). It appears that signals transduced by trivalent anti-CD3 CSA but not bivalent anti-CD3 antibodies reach the threshold to activate downstream TCR signaling molecules, possibly ERK, to upregulate FasL expression.

h145CSA characterization was performed in healthy C57BL/6 and BALB/c mice for potential wider application, and disease-prone NOD mice were used for testing its clinical efficacy. A pharmacokinetic study showed that h145CSA has a serum half-life of 11.2 h, similar to that of the F(ab’)_2_ fragment (37, 38). h145CSA is widely distributed in organs including the spleen, thymus, bone, and small intestine, among others, after intravenously injected. Administration of a 50-μg dose of h145CSA significantly reduced CD4^+^ and CD8^+^ T cells in the circulation of naïve mice within 1 h after injection. Mice receiving five doses of h145CSA reduced the numbers of conventional CD4^+^, CD8^+^ T, and Treg cells in the spleen as efficiently as their bivalent counterparts on day 3 after the last injection, although it was less efficient than others on day 1. Interestingly, h145CSA treatment allowed CD4^+^ T-cell recovery as early as day 8 after treatment, a favorable characteristic that may avoid long-lasting immunosuppression. We compared the h145CSA to other bivalent antibodies and found that a single dose of h145CSA is comparable to h145F(ab’)_2_ with minimal cytokine production and slight elevation of TNF at 2 h after administration. However, neither of them caused body weight loss. Collagen-like peptide (Gly-Pro-Pro)_10_ has been tested for its immunogenicity in rabbits and in a CD4^+^ T-cell proliferation assay with CD8^+^ T-cell-depleted human peripheral blood mononuclear cells. Both tests proved the peptide to be low-null immunogenic (unpublished data). Tissue penetration, early CD4^+^ T-cell recovery, low-to-minimum cytokine induction, and nonimmunogenic nature of (Gly-Pro-Pro)_10_ are characteristics that favor h145CSA for *in vivo* use.

T1D is a T-cell-mediated autoimmune disease. The destruction of β-cells in the pancreas is mediated by Th1 cell-related cytokines and chemokine-recruited immune cells. Adoptive transfer of Th1 cells expressing diabetogenic T-cell receptors but not their Th2-cell counterparts to neonatal NOD mice elicited diabetes 1 week after transfer (46). T cells from T1D patients stimulated with insulin-derived autoantigenic peptides exhibit Th1 phenotype and secrete IFN-γ (47). T cells from nondiabetic individuals, however, secrete IL-10 when stimulated with the same panel of peptides (47). *In vitro* studies showed that Th1 cytokine IFN-γ together with IL-1β produced by activated macrophages with or without the presence of TNF cause islet β-cell apoptosis in a nitric oxide-independent manner (48, 49). Thus, Th1 cells are crucial in the pathogenesis of T1D. You et al. transferred diabetogenic CD4^+^CD25^−^CD62L^−^ T cells or total splenocytes from NOD mice at the time before or after disease onset to NOD-SCID mice (50). They found that NOD-SCID mice receiving splenocytes from 6-week-old NOD mice developed diabetes 11 weeks after transfer, and those receiving diabetogenic CD4^+^CD25^−^CD62L^−^ T cells from mice of the same age developed disease 5 weeks after transfer. The delay in diabetes onset in the total splenocyte recipients was due to the presence of CD4^+^CD25^+^ regulatory T cells in the splenocyte population. In the same study, they also showed that CD4^+^CD25^+^ cells from 6-week-old NOD mice were more potent than cells from diabetic NOD mice in inhibiting diabetogenic CD4^+^CD25^−^CD62L^−^ T-cell proliferation. On the contrary, CD4^+^CD25^−^CD62L^−^ T cells from diabetic NOD mice were resistant to the inhibition by CD4^+^CD25^+^ cells from 6-week-old NOD mice. Their study demonstrated that the presence of regulatory T cells delays disease onset, yet the function of regulatory T cells declines and diabetogenic CD4^+^ T cells become resistant to regulatory T cells after disease onset (50). Thus, directly depleting pathogenic Th1 cells with anti-CD3 antibodies is a viable approach to treating autoimmune diabetes.

Penaranda et al. reported that treatment with FcR reduced-binding anti-CD3 antibody induces a transient increase of the percentage but not the absolute number of Treg cells due to selective depletion of conventional CD4^+^ T cells (5). They showed evidence that these Treg cells were from preexisting Treg cells and not from the expansion or conversion of conventional CD4^+^ T cells. Interestingly, the percentage of Treg cells expressing the transcription factor Helios increased after anti-CD3 antibody treatment. Helios expression could potentially stabilize Treg cell function (5). In our study, NOD mice at the time of disease onset were treated with h145CSA or h145chIgGAA for five consecutive days. Similar to what was reported by Penaranda et al., h145CSA treatment did not change the percentage of CD4^+^FoxP3^+^ Treg cells in pancreatic and mesenteric lymph nodes. Rather, the treatment significantly reduced the percentage of CD4^+^IFN-γ^+^ Th1 cells as compared to the h145chIgGAA treatment. It is probable that h145CSA being superior to h145chIgGAA in the reversal of diabetes is due to its efficiency in reducing pathogenic CD4^+^IFN-γ^+^ T cells and increasing the Treg/Th1 ratio without altering the percentage of Treg cells. It still awaits clarification whether h145CSA treatment changes the quality of Treg.

One possible drawback of CSA, as it shares with other anti-CD3 therapeutic antibodies, is that it targets nonantigen-specific CD3/TCR and may cause immunosuppression. It is of interest to note that there seems to be a discrepancy between conventional CD4^+^ T cells and CD4^+^IFN-γ^+^ Th1 cells in their susceptibility to h145CSA. The effect of h145CSA in healthy mice is transient with faster CD4^+^ T-cell recovery kinetics compared to other antibodies. However, in recent-onset diabetic NOD mice, the effect of h145CSA is long-lasting but not transient. Responder mice could maintain normoglycemia for as long as 84 days after the start of treatment. h145CSA’s ability to reduce Th1 cells in recent-onset diabetic NOD mice and to facilitate rapid recovery of conventional CD4^+^ T cells may be its advantage over other antibodies in avoiding long-lasting bystander immunosuppression.

Recent clinical trials demonstrated the role of regulatory/exhausted CD8^+^ T cells in diabetic patients responding to anti-CD3 antibody treatment. Biskirska et al. discovered an increase in the number of peripheral blood CD8^+^ T cells in T1D patients who responded to teplizumab (6). They further demonstrated that the treatment induced regulatory CD8^+^CD25^+^ cells that express CTLA4 and FoxP3, which inhibit the responses of CD4^+^ T cells. A recent study showed that administration of foralumab intranasally reduced CD8^+^ effector memory cells and granzyme B and perforin expression (51). Foralumab induced regulatory phenotype TIGIT, TGFB1, and KIR3DL2 in the CD8^+^ TEMRA population (51). Exhausted CD8^+^ T cells, including those with the phenotypes of CD57^-^KLRG1^+^PD-1^+^, TIGIT^+^KLRG1^+^, and persistent downmodulated CD127, were found in patients treated with teplizumab (7, 52). The presence of these cells was associated with a favorable response to teplizumab treatment (7, 52). In our study, multiple injections of h145CSA reduced the number of CD8^+^ T cells as efficiently as bivalent antibodies h145chIgGAA and h145F(ab’)_2_ in naïve animals. Since the efficacy of h145CSA is significantly greater than h145chIgGAA in treating diabetes, we speculate that even if regulatory CD8^+^ T cells were induced after treatment with either antibody, they may not contribute to the differential efficacy between h145CSA and h145chIgGAA treatments of NOD mice after onset of diabetes.

Teplizumab has been approved for the treatment of individuals at high risk for T1D. The trial conducted between July 2011 and November 2018 showed that it successfully delays the progression to clinical T1D in high-risk individuals (27). Thus, the use of FcR reduced-binding anti-CD3 antibody to treat T1D is a promising approach. Five injections of anti-CD3 CSA at 25 μg per dose efficiently reduce Th1 cells and are efficacious in inducing long-lasting remission in one-third of recent-onset NOD mice. With anti-CD3 CSA having the advantage of low-minimum cytokine inducibility and low immunogenicity in the animal host, it is possible to increase the injection dose and improve its efficacy. Thus, anti-CD3 CSA possesses the characteristics that have the potential for clinical use in the treatment of T1D.

## Data availability statement

The original contributions presented in the study are included in the article/**Supplementary Material**. Further inquiries can be directed to the corresponding authors.

## Ethics statement

The animal study was reviewed and approved by Industrial Technology Research Institute (permit: ITRI-IACUC-2010-050M, ITRI-IACUC-2019-070, ITRI-IACUC-2019-071, ITRI-IACUC-2021-043); The Jackson Laboratory (JAX-13060); National Laboratory Animal Center, National Applied Research Laboratories, Taiwan (NLAC(TN)-110-M-007, NLAC(TN)-111-M-001, NLAC-111-M-022); Center of Toxicology and Preclinical Sciences at the Development Center for Biotechnology, Taiwan (DCB-2010-TP-001); Institute of Nuclear Energy Research, Taiwan (# 99064).

## Author contributions

C-CH: conceived, designed, and performed experiments; analyzed data; and drafted and finalized manuscript. H-HS: performed experiments. H-CL: performed and analyzed SEC-HPLC experiment. S-CM: designed and provided materials for T-cell differentiation experiments. JK: designed experiment and reviewed and edited the manuscript. M-YC: conceived, designed, and supervised experiments; acquired funding; and reviewed, edited, and finalized the manuscript. BW-H: conceived, designed, and supervised experiments; validated data analysis; and drafted, edited, and finalized the manuscript. All authors listed have made a substantial, direct, and intellectual contribution to the work and approved the manuscript for publication.
