# Supplementary material for: A novel trivalent non-Fc anti-CD3 Collabody preferentially induces Th1 cell apoptosis in vitro and long-lasting remission in recent-onset diabetic NOD mice
Source: Front Immunol. 2023 Aug 3;14:1201853. doi: 10.3389/fimmu.2023.1201853 (PMC10435756; doi:10.3389/fimmu.2023.1201853)

Supplementary Material

A novel trivalent non-Fc anti-CD3 Collabody preferentially induces Th1 cell apoptosis *in vitro* and long-lasting remission in recent-onset diabetic NOD mice

Chuan-Chuan Huang[1,2], Hsiang-Hsuan Sung[3], Hsiu-Chuan Li[2], Shi-Chuen Miaw[1], John T. Kung[4], Min-Yuan Chou[2*], Betty A. Wu-Hsieh[1*]

- **Correspondence:** Betty A. Wu-Hsieh, bwh@ntu.edu.tw ;

Min-Yuan Chou, minyuanc@itri.org.tw

Supplementary Figures

Supplementary Figure 1

Purification and structural characterization of anti-CD3 antibodies. Whole immunoglobulins and h145CSA were expressed as soluble secretory proteins in CHO-S cells and purified from culture media by column chromatography. The purity of these antibodies was analyzed by SDS-PAGE **(A)** and SEC-HPLC **(B)**. **(A)** Indicated antibodies were electrophoresed on a 4-12% SDS/Bis-Tris polyacrylamide gel with MES buffer under non-reducing (-DTT, lanes 1 to 4) and reducing (+DTT, lanes 5 to 8) conditions. The gel was stained with Coomassie blue. **(B)** Chromatograms of the indicated antibodies were analyzed using a SEC-HPLC column. Arrows point to the monomeric form of each antibody variant and the corresponding purity value (%) is shown in parentheses.

Supplementary Figure 2

Purified T cells were incubated with 10-fold serial dilutions of each anti-CD3 antibody variants at 37°C for 24 h. Cells were stained with either Alexa Fluor 647-conjugated anti-CD4 and PE-conjugated anti-TCRβ antibody or with Alexa Fluor 647-conjugated anti-CD8 and PE-conjugated anti-TCRβ antibody, followed by flow cytometric analysis. % TCR downregulation = the MFI difference of TCR on the surface between antibody-treated and antibody-untreated T cells divided by the MFI of TCR on the surface of untreated T cells × 100. The results are expressed as the mean of triplicate experiments ± SEM.

Supplementary Figure 3

Phenotypic characterization of in vitro-differentiated Th1 and Treg cells. (A, B) Spleens from healthy female C57BL/6 mice at 6-12 weeks of age were collected. Naïve splenic $CD4^+$ T cells were enriched by using Mouse Naïve CD4 T cell Isolation Kit and subject to Th1 or Treg skewing condition. Th1 cells were exposed to 50 ng/ml of PMA, 500 ng/ml of Ionomycin and 10 μg/mL of Brefeldin A for 4 h before staining with Alexa Fluor 488-conjugated anti-CD4 and Alexa Fluor 647-conjugated anti-IFN-γ or Alexa Fluor 647-conjugated isotype control antibodies. Treg cells were stained with Alexa Fluor 647-conjugated anti-CD4 and PE-conjugated anti-FoxP3 or PE-conjugated isotype control antibodies. Cells were subject to flow cytometric analysis. Dot plots show the % of $CD4^+IFN\text{-}\gamma^+$ cells (A) and $CD4^+FoxP3^+$ cells (B) in total $CD4^+$ T cell population cultured in Th1 or Treg skewing condition, respectively. One representative of three independent experiments is shown.

Supplementary Figure 4

Spleens from healthy female C57BL/6 mice at 6-12 weeks of age were collected. Naïve splenic $CD4^+$ T cells were enriched by using Mouse Naïve CD4 T cell Isolation Kit and subject to Th1 skewing conditions. Th1 cells were exposed to 10 μg/ml of 145-2C11, h145chIgGAA, h145CSA, or medium alone for 6, 12, and 24 h before staining with FITC-conjugated Annexin V and 7-AAD. Cells were subject to flow cytometric analysis. Percent Annexin V^+ cells are recorded. Data were pooled from three independent experiments. The bars represent the mean ± SEM. Data were analyzed by two-way ANOVA followed by Tukey post-hoc test. ****$p < 0.0001$.

Supplementary Figure 5

Anti-CD3 antibody treatments do not change Fas expression and the levels of *Bcl2* and *Bax* in Th1 cells. *In vitro* differentiated Th1 cells were treated with 145-2C11, h145chIgGAA, h145CSA or without treatment. **(A)** Cells were harvested 6, 12, and 24 h later and stained with PE-conjugated anti-Fas antibody and analyzed by flow cytometry. Histograms show the fluorescence intensity of Fas on cells. Data shown are from one representative of three independent experiments. **(B, C)** *In vitro* differentiated Th1 cells were treated with 10 μg/ml of either 145-2C11, h145chIgGAA or h145CSA for 3 h and 6 h or without treatment. Treated cells were subject to mRNA extraction. *Bcl2* **(B)** and *Bax* **(C)** mRNA expression were analyzed by RT-qPCR and normalized against *Actb* mRNA. Relative expression level of *Bcl2/Bax* was calculated as: normalized *Bcl2/Bax* mRNA

expression of cells with antibody treatment divided by normalized *Bcl2/Bax* mRNA expression of cells in medium alone at the same time point. Each symbol represents datum from one independent experiment. Data were analyzed by two-way ANOVA followed by Tukey post-hoc test. ns, not significant.

Supplementary Figure 6

h145CSA treatment induces up-regulation of FasL in Th1 cells. Spleens from healthy female C57BL/6 mice at 6-12 weeks of age were collected. Naïve splenic $CD4^+$ T cells were enriched by using Mouse Naïve CD4 T cell Isolation Kit and subject to Th1 or Treg skewing conditions. In vitro differentiated Th1 (**A**) and Treg (**B**) cells were treated with 10 μg/ml of 145-2C11, h145chIgGAA, h145CSA or cultured in medium alone. Cells were cultured for 6, 12, and 24 h before staining with biotin-conjugated anti-FasL antibody followed by APC-conjugated Streptavidin and subject to flow cytometric analysis. The dot plots of FSC and SSC and histograms of APC-conjugated Streptavidin (FasL) of total cells are shown. % of total cells expressing FasL on cell surface are shown on the upper right corner and MFI of APC signals are shown on the histograms. One representative of four independent experiments is shown.

Supplementary Figure 7

Pharmacokinetics and biodistribution profile of h145CSA in mice. **(A)** BALB/c mice were injected intravenously with h145CSA at 1 mg/kg of body weight. Blood samples were collected at different time points. The levels of h145CSA in plasma were determined by ELISA using HRP-conjugated goat anti-human kappa light chain antibody. Results are the mean of samples from 3 animals for each time point; error bars represent SD. Pharmacokinetic parameters were calculated by two-compartmental pharmacokinetic analysis using PKSolver 2.0. **(B)** BALB/c mice were injected with 25 μg of ^{131}I-h145CSA intravenously. The organs were excised 0.5, 1, 2, 6, 24 or 48 h later, weighed and the ^{131}I activity was measured. Five mice were included in each group. The radioactivity of different tissues is expressed as percentage of injection dose per gram (% ID/g) which was calculated by dividing the radioactivity per gram of tissue by the initial radioactivity count before injecting into the mice. **(C)** The tissue-to-blood ratio of radioactivity was obtained by dividing the radioactivity counted of each tissue (% ID/g) by that of blood. Data were expressed as mean ± SEM. S.I.: small intestine

Supplementary Figure 8

Dose titration of h145CSA in naïve C57BL/6 mice. **(A)** Naïve C57BL/6 mice were intraperitoneally injected with h145CSA ranging from 0.03 to 50 μg per mouse. Mice were bled at 2 h after administration. Peripheral blood cells were stained with Alexa Fluor 488-conjugated anti-CD4, PE-conjugated anti-CD8 and PerCP/Cy5.5-conjugated anti-CD45 antibodies and subject to flow cytometric analysis. The numbers of circulating $CD4^+$ and $CD8^+$ T cells in each mouse were counted. **(B)** Mice were intraperitoneally given 25 or 50 μg of h145CSA and bled at 1 and 2 h after administration. The numbers of circulating $CD4^+$ and $CD8^+$ T cells were compared. Each symbol represents one mouse. n = 5, for all groups except for 1 h datum point for mice receiving 25 μg (n = 10). Data were analyzed by two-way ANOVA followed by Sidak multiple comparisons post-hoc test. *$p < 0.05$, **$p < 0.01$; ns, not significant.

Supplementary Figure 9

h145CSA at 0.1, 1, and 10 μg/ml is more efficient than bivalent anti-CD3 antibodies in inducing Th1 cell apoptosis. Th1 cells were generated from *in vitro* differentiation. Cells were exposed to 0.1, 1, and 10 μg/ml of 145-2C11, h145chIgGAA, h145CSA, or medium alone for 6, 12, and 24 h before staining with FITC-conjugated Annexin V and 7-AAD. Cells were subject to flow cytometric analysis. Percent specific apoptotic cells were calculated as: (Δ Annexin V % = % Annexin V^+ cells with anti-CD3 antibody treatment – % Annexin V^+ cells in medium alone). Data were pooled from three independent experiments. The bars represent the mean ± SEM. Data were analyzed by two-way ANOVA followed by Tukey post-hoc test. ***$p < 0.001$, ****$p < 0.0001$.

Supplementary Figure 1

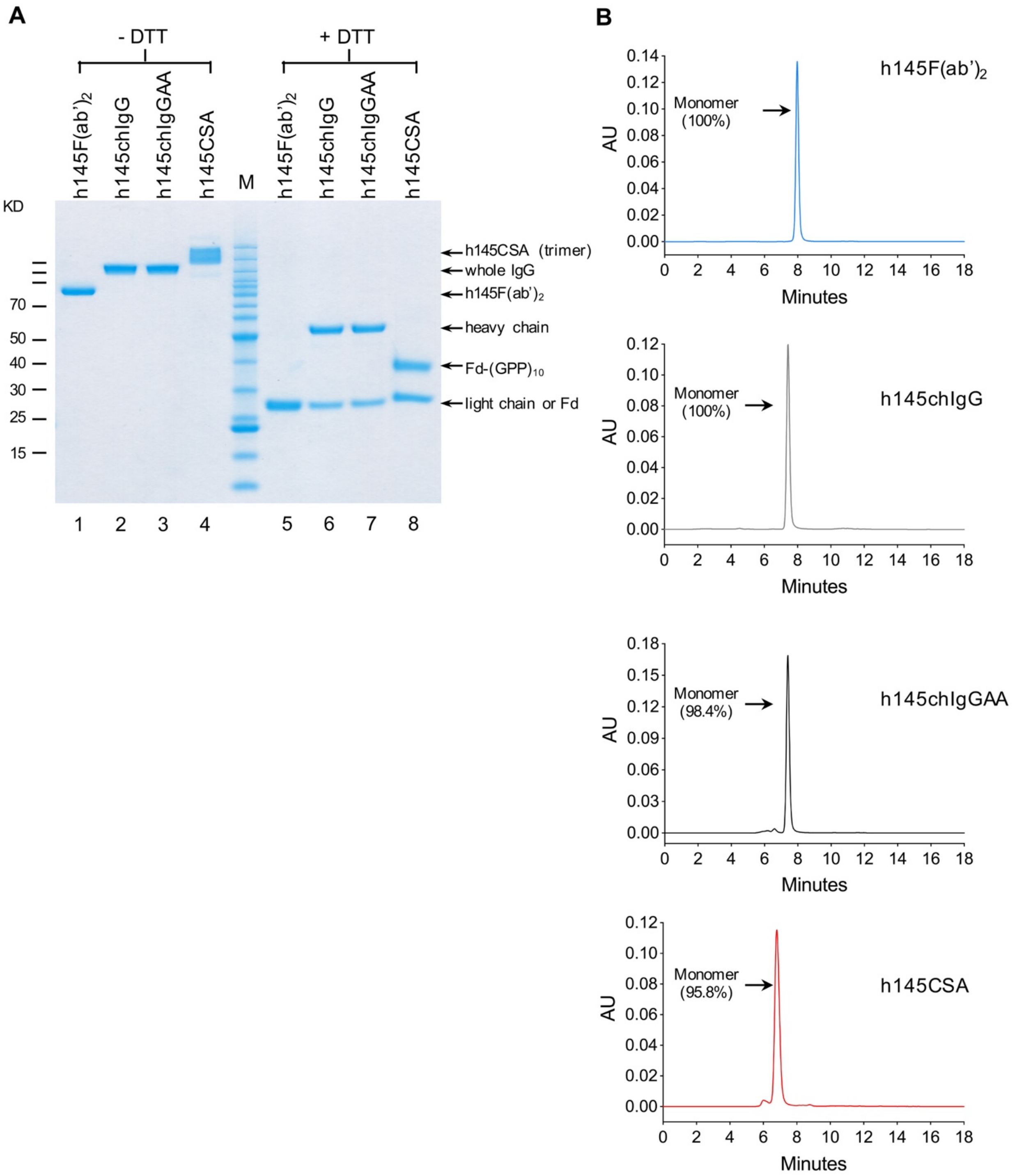

Supplementary Figure 2

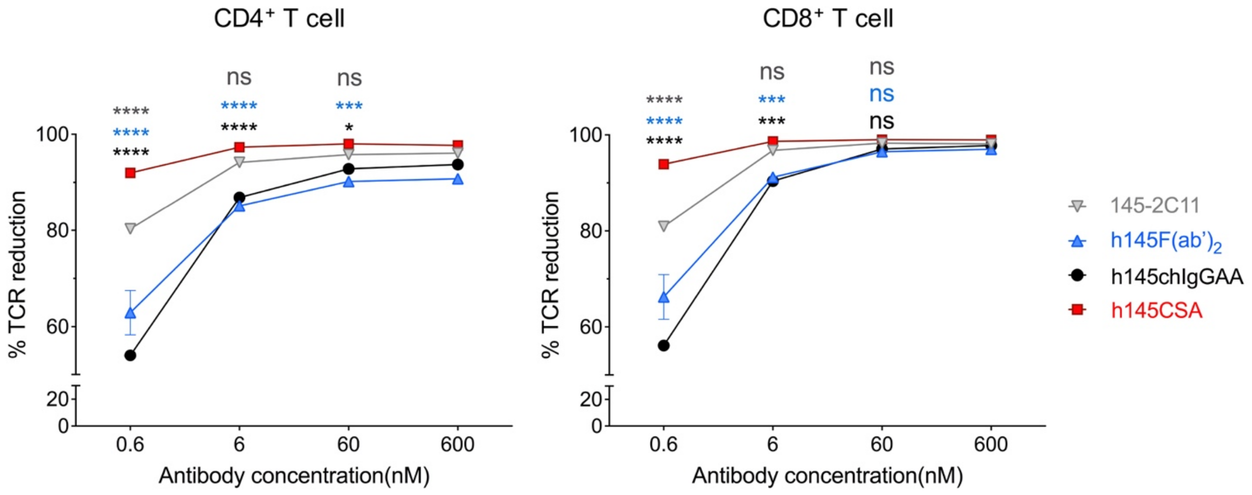

Supplementary Figure 3

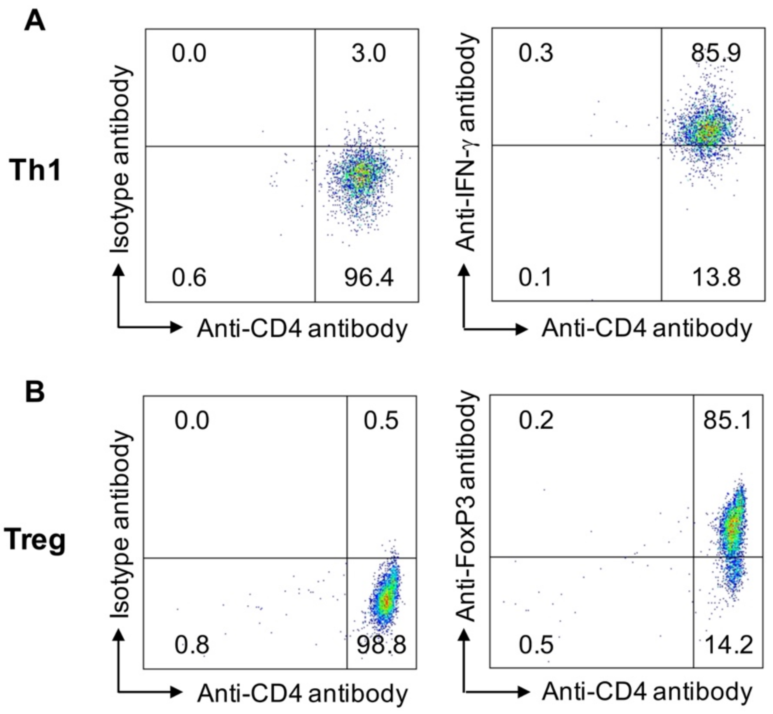

Supplementary Figure 4

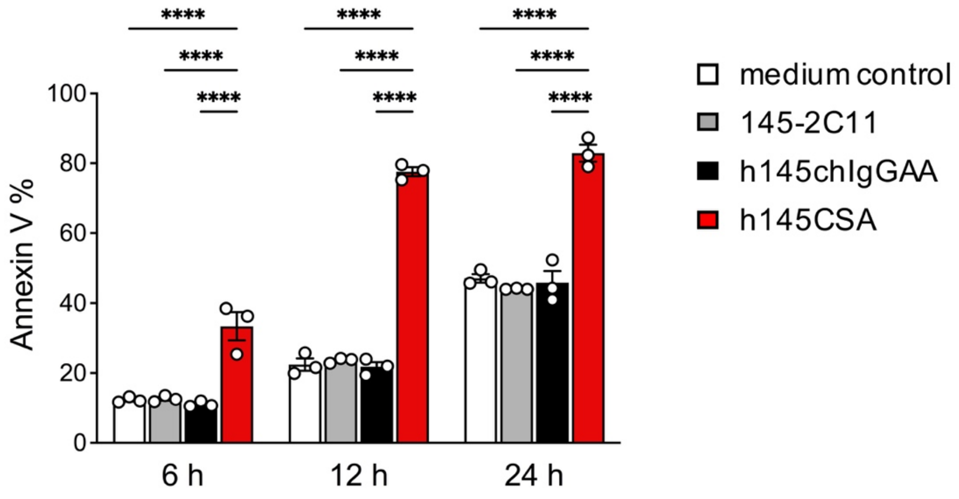

Supplementary Figure 5

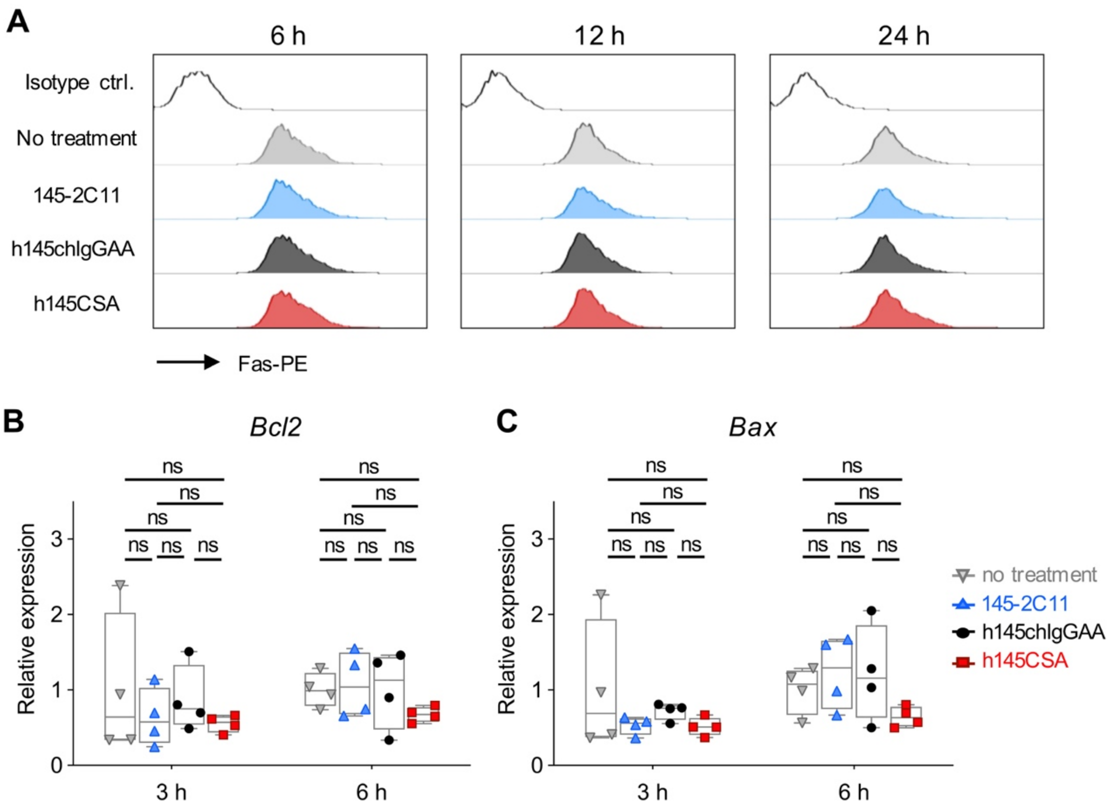

Supplementary Figure 6

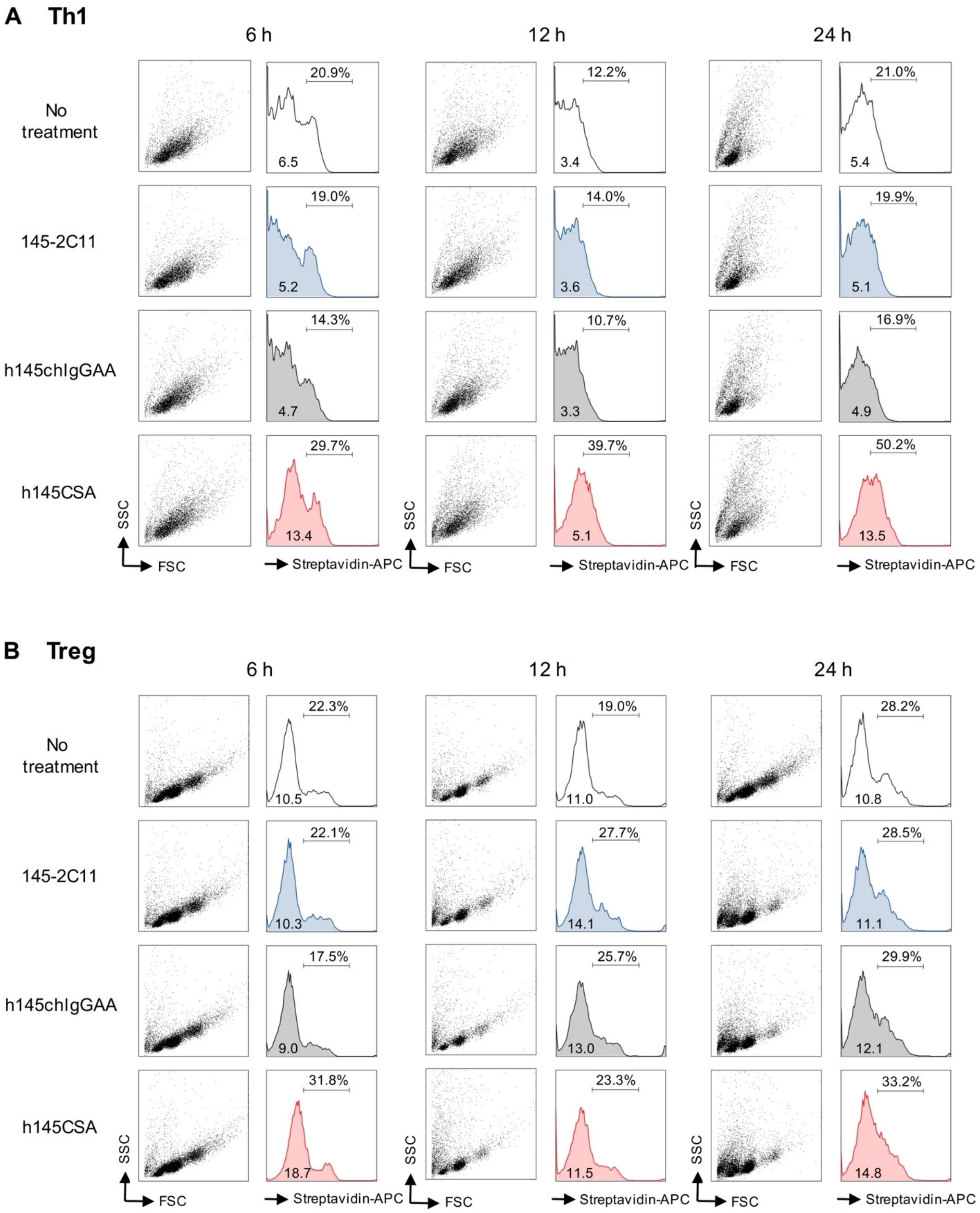

Supplementary Figure 7

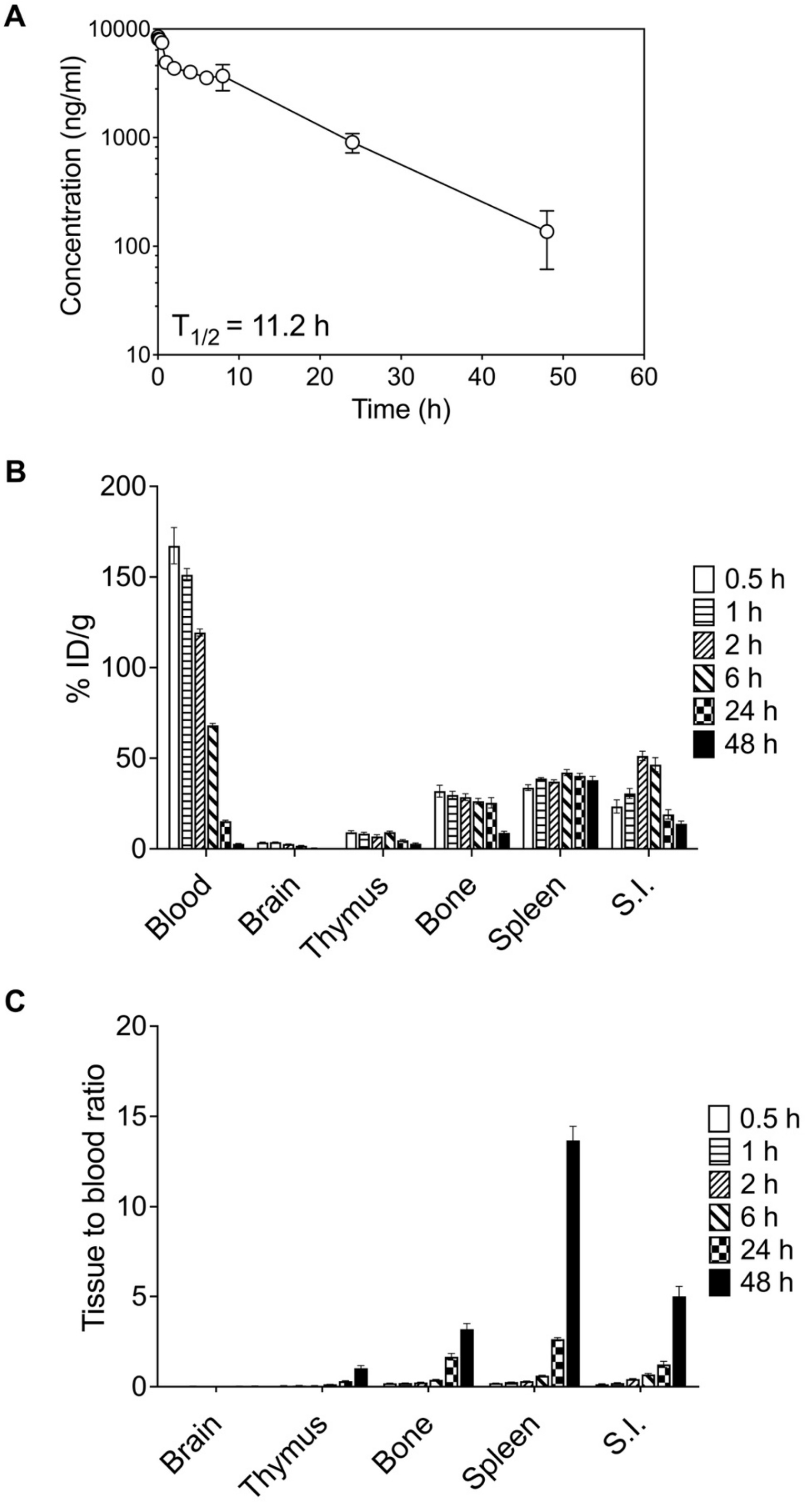

Supplementary Figure 8

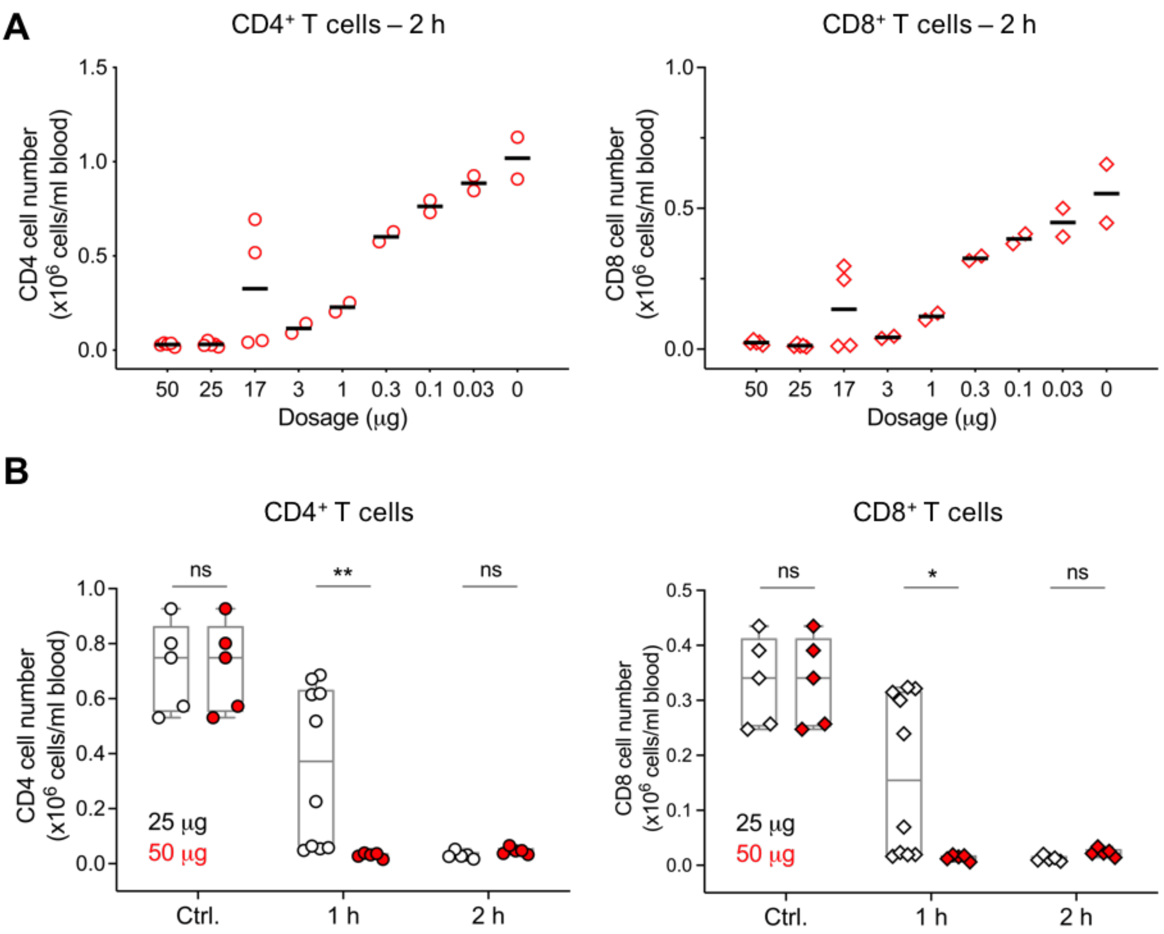

Supplementary Figure 9

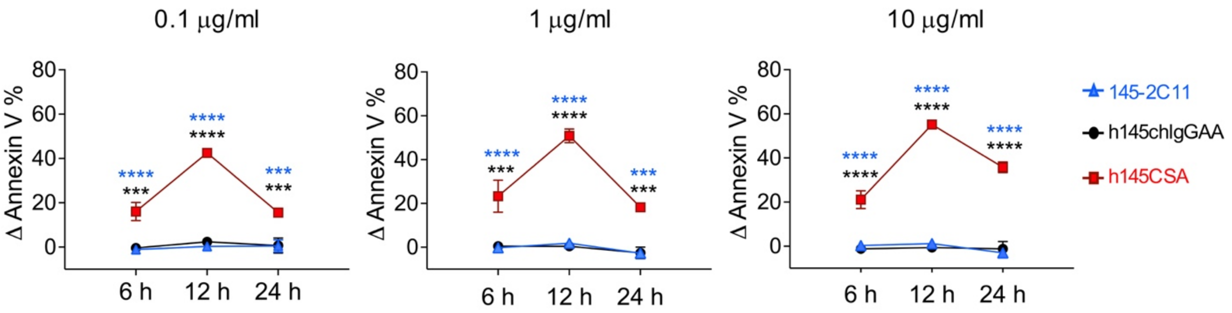

Supplement: Supplementary file 1 [file DataSheet_1.pdf]
