# Supplementary figures and images for: A novel trivalent non-Fc anti-CD3 Collabody preferentially induces Th1 cell apoptosis in vitro and long-lasting remission in recent-onset diabetic NOD mice
Source: Front Immunol. 2023 Aug 3;14:1201853. doi: 10.3389/fimmu.2023.1201853 (PMC10435756; doi:10.3389/fimmu.2023.1201853)

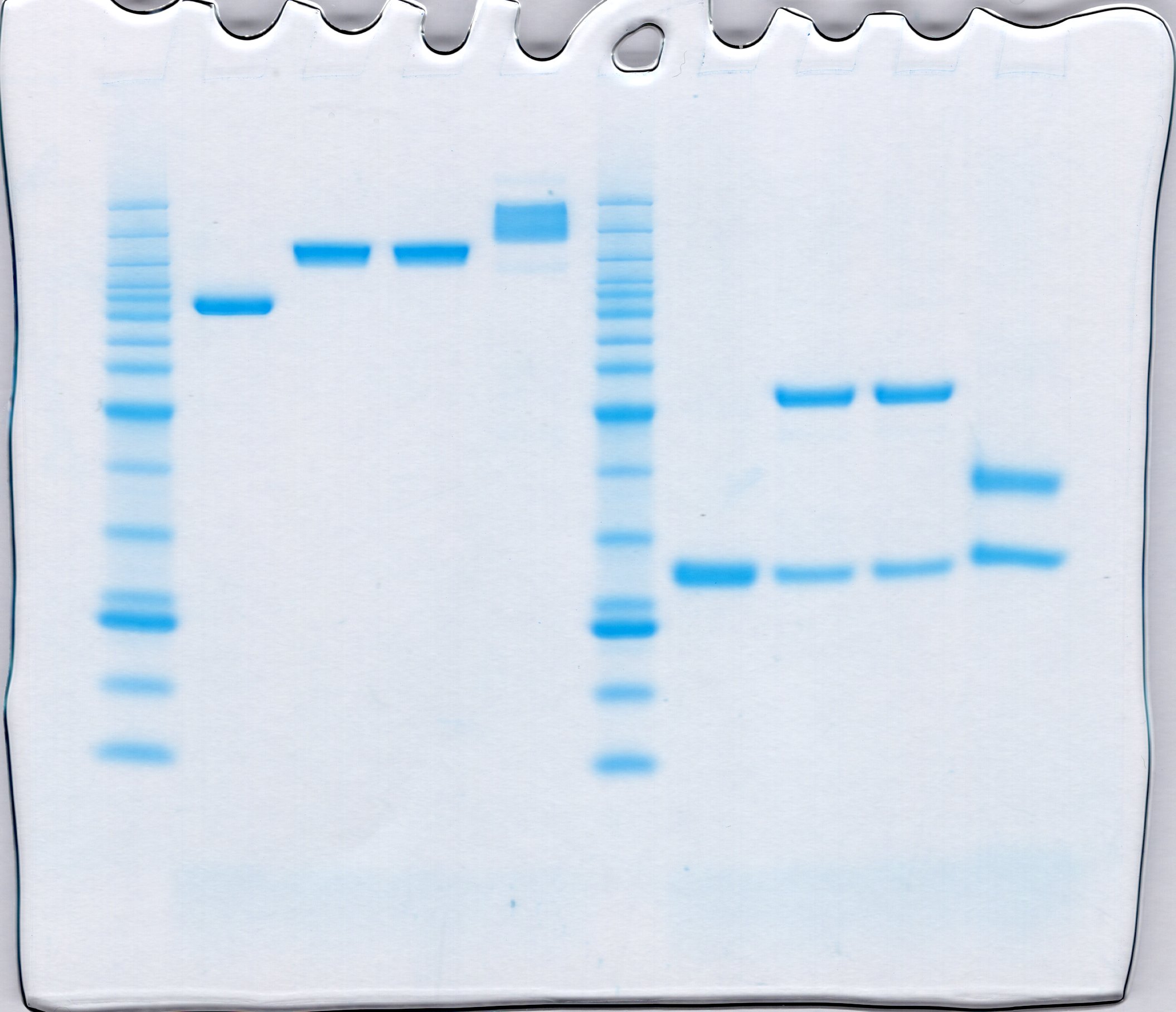

Supplement: Supplementary file 9 [file Image_1.jpeg]
